# LRR-protein RNH1 dampens the inflammasome activation and is associated with COVID-19 severity

**DOI:** 10.26508/lsa.202101226

**Published:** 2022-03-07

**Authors:** Giuseppe Bombaci, Mayuresh Anant Sarangdhar, Nicola Andina, Aubry Tardivel, Eric Chi-Wang Yu, Gillian M Mackie, Matthew Pugh, Vedat Burak Ozan, Yara Banz, Thibaud Spinetti, Cedric Hirzel, Esther Youd, Joerg C Schefold, Graham Taylor, Amiq Gazdhar, Nicolas Bonadies, Anne Angelillo-Scherrer, Pascal Schneider, Kendle M Maslowski, Ramanjaneyulu Allam

**Affiliations:** 1 Department of Hematology and Central Hematology Laboratory, Inselspital, Bern University Hospital, University of Bern, Bern, Switzerland; 2 Department for BioMedical Research, University of Bern, Bern, Switzerland; 3 Graduate School for Cellular and Biomedical Sciences, University of Bern, Bern, Switzerland; 4 Department of Biochemistry, University of Lausanne, Lausanne, Switzerland; 5 Institute of Immunology and Immunotherapy, University of Birmingham, Birmingham, UK; 6 Institute of Metabolism and Systems Research, University of Birmingham, Birmingham, UK; 7 Institute of Pathology, University of Bern, Bern, Switzerland; 8 Department of Intensive Care Medicine, Inselspital, Bern University Hospital, University of Bern, Bern, Switzerland; 9 Department of Infectious Diseases, Inselspital, Bern University Hospital, University of Bern, Bern, Switzerland; 10 School of Medicine, Dentistry and Nursing, Forensic Medicine and Science. University of Glasgow, Scotland, UK; 11 Department of Pulmonary Medicine, Inselspital, Bern University Hospital, University of Bern, Bern, Switzerland

## Abstract

Inflammasomes are cytosolic innate immune sensors of pathogen infection and cellular damage that induce caspase-1–mediated inflammation upon activation. Although inflammation is protective, uncontrolled excessive inflammation can cause inflammatory diseases and can be detrimental, such as in coronavirus disease (COVID-19). However, the underlying mechanisms that control inflammasome activation are incompletely understood. Here we report that the leucine-rich repeat (LRR) protein ribonuclease inhibitor (RNH1), which shares homology with LRRs of NLRP (nucleotide-binding oligomerization domain, leucine-rich repeat, and pyrin domain containing) proteins, attenuates inflammasome activation. Deletion of RNH1 in macrophages increases interleukin (IL)-1β production and caspase-1 activation in response to inflammasome stimulation. Mechanistically, RNH1 decreases pro-IL-1β expression and induces proteasome-mediated caspase-1 degradation. Corroborating this, mouse models of monosodium urate (MSU)-induced peritonitis and lipopolysaccharide (LPS)-induced endotoxemia, which are dependent on caspase-1, respectively, show increased neutrophil infiltration and lethality in *Rnh1*^−/−^ mice compared with wild-type mice. Furthermore, RNH1 protein levels were negatively related with disease severity and inflammation in hospitalized COVID-19 patients. We propose that RNH1 is a new inflammasome regulator with relevance to COVID-19 severity.

## Abstract (precis)

RNH1 prevents inflammation by inhibiting inflammasome activation through controlling caspase-1 protein levels. In COVID-19 patients, RNH1 expression levels were negatively associated with disease severity and inflammation, suggesting a role for RNH1 in SARS-CoV-2–mediated inflammation and pathology.

## Introduction

Ribonuclease inhibitor (RNH1) is a cytosolic leucine-rich repeat (LRR) protein; however, it can also be found in the nucleus and mitochondria (Dickson et al, 2005; Furia et al, 2011). The known function of RNH1 is to bind to pancreatic-type ribonucleases with femtomolar affinity and render them inactive (Dickson et al, 2005). In addition, RNH1 has been shown to inhibit oxidative damage, regulate angiogenin (ANG)-mediated neovascularization, and prevent tiRNAs (tRNA-derived stress-induced RNAs) production (Monti et al, 2007; Dickson et al, 2009; Yamasaki et al, 2009). RNH1 has also been shown to bind and inhibit ER stress sensor IRE1 (inositol-requiring enzyme 1) (Tavernier et al, 2018). However, the relevance of these observations in vivo has yet to be validated. We recently reported that RNH1 is important for embryonic erythropoiesis in mice, suggesting possible additional roles for RNH1 in mammalian physiology (Chennupati et al, 2018). RNH1 was the first cytosolic protein identified to contain LRRs (Dickson et al, 2005). LRRs are present in a large family of proteins that display vast surface areas to foster protein–protein and protein–ligand interactions (Kobe & Deisenhofer, 1995). LRR proteins are classified into subfamilies based on the organism of origin, cellular localization, LRR consensus sequence and length. To date, seven LRR subfamilies of proteins have been described: bacterial, ribonuclease inhibitor (RNH1)–like, cysteine-containing, SDS22, plant-specific, typical, and small (Kobe & Kajava, 2001; Dickson et al, 2005; Matsushima et al, 2010). The members of the RNH1-like subfamily are intracellular proteins and include human MHC class II transactivator (CIITA), Ran GTPase–activating protein from *Saccharomyces pombe* (RANGAP1), and other NOD-like receptors (NLRs) (Dickson et al, 2005). Interestingly, the LRRs of RNH1 are very similar to those of NLRP (nucleotide-binding oligomerization domain, LRR and pyrin domain containing) proteins (Ng et al, 2011) which form inflammasome complexes.

Inflammasomes are cytosolic pattern recognition receptors, which sense invading pathogens or endogenous danger molecules that are released by dying cells. This process is critical in eliminating pathogens and initiating tissue repair (Gong et al, 2020). Inflammasomes form multiprotein caspase-1–activating complexes upon activation to mediate inflammatory response. Inflammasome complexes contain sensors (e.g., NLRP3, nucleotide-binding oligomerization domain, LRR, and pyrin domain containing-3), an adaptor protein ASC (apoptosis-associated speck-like protein containing a caspase activation and recruitment domain), and effector protein caspase-1 (Martinon et al, 2009; Schroder & Tschopp, 2010). Assembly of these components into an inflammasome is initiated after sensing several host-derived danger-associated molecular patterns, bacterial toxins, nucleic acids, pathogenic crystals, and altered cellular components (Henao-Mejia et al, 2014; Gong et al, 2020). An active inflammasome complex catalyzes proteolytic cleavage of the pro-caspase-1 protein into functional caspase-1. Subsequently, caspase-1 processes IL-1 cytokine members pro-IL-1β and pro-IL-18 into biologically active IL-1β and IL-18 and initiates gasdermin-D (GSDMD)–mediated pyroptosis, a form of cell death (Broz & Dixit, 2016). Several NLR proteins (NLRP1, NLRP3, NLRP6, and NLRC4/NAIP), HIN200 proteins (AIM2), and pyrin can form inflammasome complexes (Schroder & Tschopp, 2010; Broz & Dixit, 2016). The NLR family is characterized by the presence of a central nucleotide-binding and oligomerization (NACHT) domain, which is commonly flanked by C-terminal LRRs and N-terminal CARD (caspase activation and recruitment domain) or PYD (Pyrin domains). LRRs are believed to function in ligand sensing and autoregulation, whereas CARD and PYD domains mediate homotypic protein–protein interactions for downstream signaling (Schroder & Tschopp, 2010).

Inflammasomes are critical to mount immune responses; however, uncontrolled inflammasome activation is associated with several autoimmune and inflammatory diseases including Coronavirus disease (COVID-19) (Strowig et al, 2012; van den Berg & Velde, 2020). COVID-19 caused by severe acute respiratory syndrome coronavirus 2 (SARS-CoV-2) is associated with significant mortality and has resulted in more than ∼5.47 million deaths worldwide as of 11 January 2022 according to World Health Organization (WHO) data report (https://covid19.who.int). The clinical manifestation of patients with COVID-19 includes hypoxemic respiratory failure, acute respiratory distress syndrome, and myocardial injury, among others (Huang et al, 2020a). Recent studies have reported increased inflammasome activation and inflammation in severe COVID-19 patients and suggest that increased inflammasome activation potentially mediates disease progression in COVID-19 (Kroemer et al, 2020; Lucas et al, 2020; Toldo et al, 2020; Pan et al, 2021; Rodrigues et al, 2021; Vora et al, 2021). It is therefore important to understand the molecules that control and resolve the overactivation of inflammasomes and inflammation to develop therapeutic targets for COVID-19 and other inflammatory disorders. Despite being homologous with LRRs of NLRPs, the role of RNH1 in inflammasome activation has not been investigated.

In this study, we found that RNH1 inhibits inflammasome activation by controlling proteasome-mediated degradation of the downstream effector molecule caspase-1. RNH1-deleted mice displayed higher inflammasome-dependent activation of caspase-1. Interestingly, we also found that RNH1 expression in buffy coat and lung biopsies is negatively related with SARS-CoV-2–mediated severity and inflammation in COVID-19 patients. Collectively, these findings establish RNH1 as a previously unidentified negative regulator of inflammasome activation and indicate its potential role in human inflammatory diseases including SARS-CoV-2–mediated inflammation and pathology.

## Results

### RNH1 is predominantly expressed in myeloid cells and is increased under inflammatory conditions

**Figure 1.**
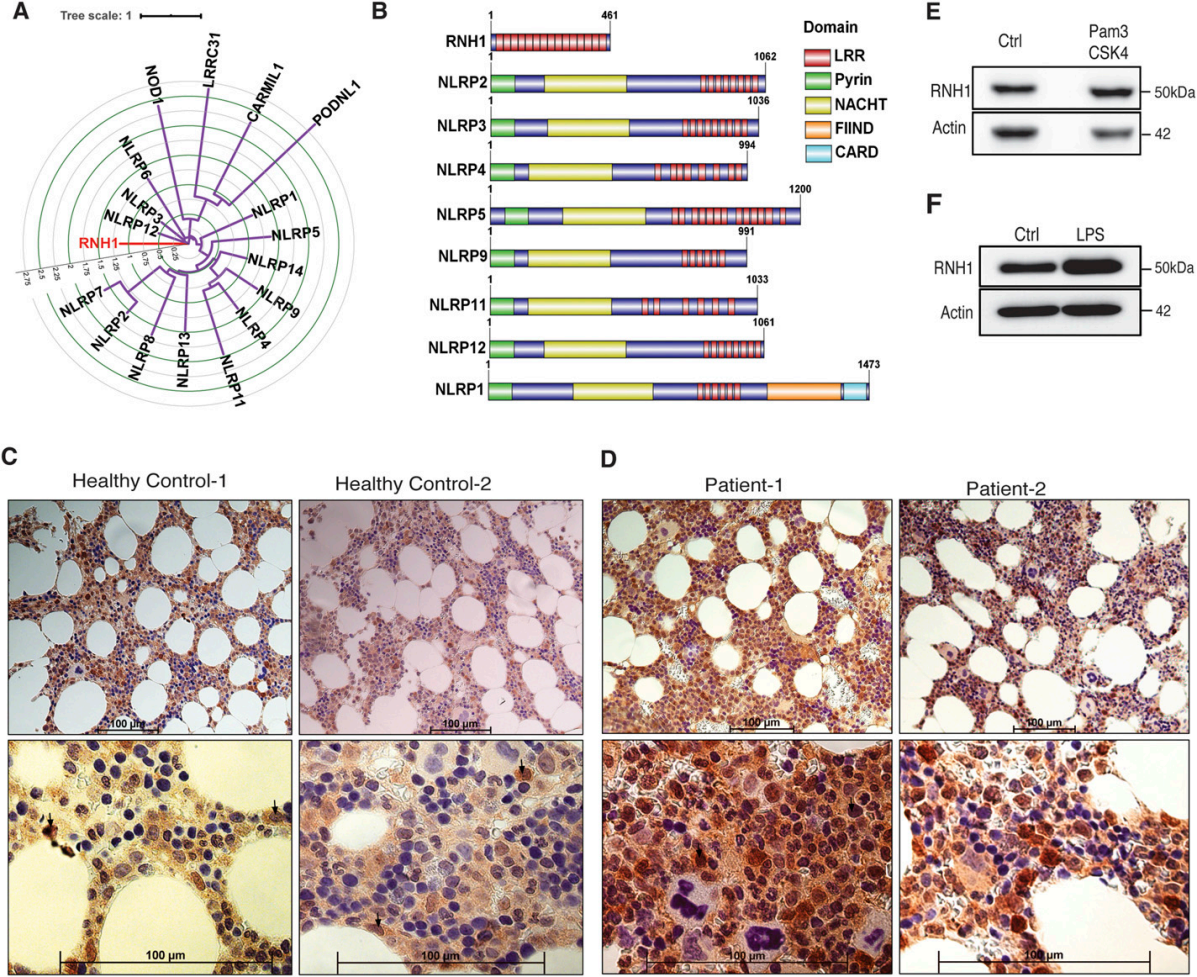
RNH1 shares homology with multiple NLR proteins and is expressed in myeloid cells. **(A)** Circular tree representing the domain conservation relationship of human RNH1. Protein sequence alignments were made using MAFFT. A maximum likelihood phylogenetic tree was generated using IQ-Tree with 1,000 bootstrap replicates. Internal tree scale is shown with circular grid. **(B)** Structural alignment of protein domains. The domain information of selected proteins taken from Uniprot and represented using Illustrator for Biological Sequences (IBS) tool. **(C)** Human healthy BM biopsies were stained with RNH1 antibody. Myeloid cells showing high RNH1 expression are indicated with arrows. Scale bar 100 μm. **(D)** BM biopsies from patients with confirmed inflammatory conditions were stained with RNH1 antibody. Myeloid cells with increased RNH1 expression are indicated with arrows. These patients were non–COVID-19 patients. Scale bar 100 μm. **(E)** THP1 cells were stimulated with TLR2 ligand Pam3CSK4 (1 μg/ml) for 24 h. Total protein lysates were isolated and analysed by Western blot with indicated antibodies. Blots are representative of two independent experiments. **(F)** Mouse primary BMDMs were stimulated with TLR4 ligand LPS (1 μg/ml) for 24 h. Total protein lysates were isolated and analysed by Western blot with indicated antibodies. Blots are representative of two independent experiments. NLRP (nucleotide-binding oligomerization domain, leucine-rich repeat and pyrin domain–containing protein), NACHT (nucleotide-binding oligomerization domain), LRR (leucine-rich repeat protein), CARD (caspase activation and recruitment domain), CARMIL1 (capping protein regulator and myosin 1 linker 1), PODN L1 (podocan like 1), LRRC31 (leucine-rich repeat containing 31), NOD1 (nucleotide-binding oligomerization domain containing 1), and FIIND (function to find domain). Source data are available for this figure.

Human RNH1 protein consists of 15 LRR repeats whose sequence and organization are similar to those present in multiple NLRP proteins (Ng et al, 2011; Hoss et al, 2019) (Fig 1A). Whereas LRRs of NLR proteins are confined to the C-terminal only, the RNH1 protein sequence consists entirely of LRRs and lacks any other identifiable functional domains (Fig 1B). LRRs in NLRP family proteins are believed to function in ligand sensing and autoregulation (Schroder & Tschopp, 2010). However, whether RNH1 is also involved in inflammation similar to NLRP proteins has not been investigated. First, we checked RNH1 expression in hematopoietic cells by staining healthy human BM biopsies for RNH1. We found that RNH1 is expressed at higher levels in myeloid cells compared with other hematopoietic cell-types such as lymphocytes and erythrocytes (Fig 1C). Furthermore, RNH1 protein expression is elevated in BM biopsies of patients with confirmed systemic inflammation (see the Materials and Methods section) (Fig 1D). Corroborating this, RNH1 protein levels were moderately increased in human monocyte cell-line (THP1 cells) upon stimulation with the TLR1/2 ligand Pam3Cysk4 (Fig 1E) and in primary mouse BMDMs after TLR4 stimulation with LPS (Fig 1F). Collectively, these results suggest that RNH1 is predominantly expressed in myeloid cells and may play a role in inflammation.

### Absence of RNH1 increases NLRP3 inflammasome activation

**Figure 2.**
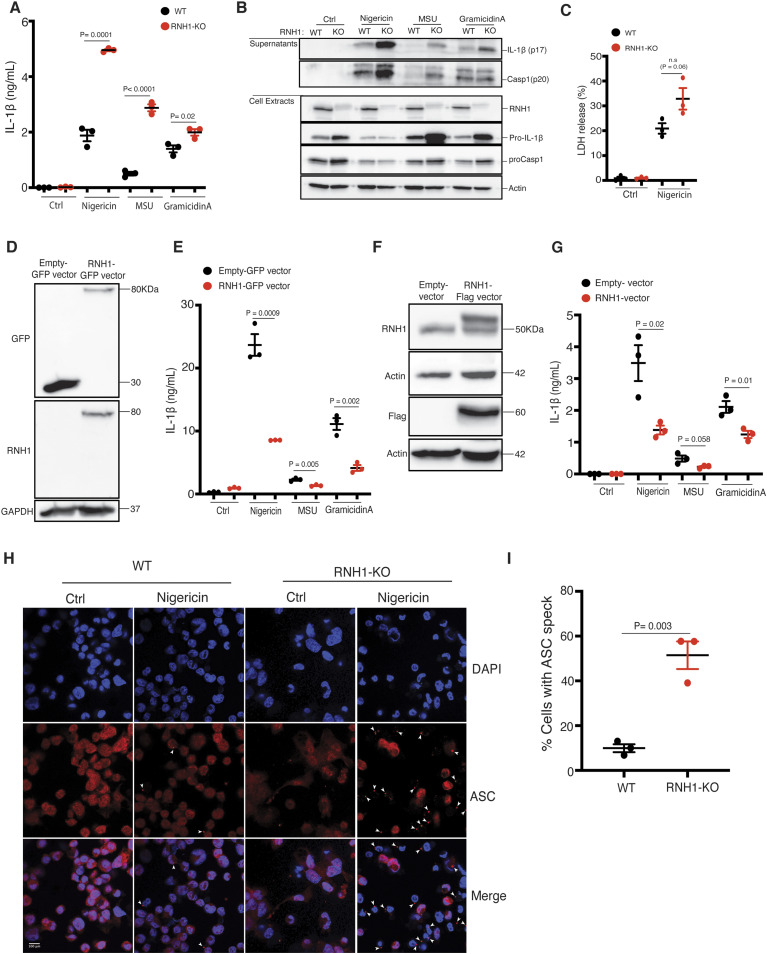
RNH1 inhibits NLRP3 inflammasome activation. **(A, B)** PMA differentiated wild-type (WT) and RNH1-KO THP1 cells were stimulated with Nigericin (5 μM) for 1 h or with MSU (500 μg) and Gramicidin A (30 μg) for 5 h. **(A)** Supernatants were collected and IL-1β ELISA was performed. Data shown as mean ± SEM of pooled data from three independent experiments. Statistical analyses were performed using a two-tailed *t* test. **(B)** Cell lysates and supernatants were analysed for pro- and cleaved-forms of caspase-1 and IL-1β by Western blot. Blots were representative of three independent experiments. **(C)** WT and RNH1-KO THP1 cells were stimulated with NLRP3 agonist Nigericin (5 μM) for 18 h. Supernatants were collected, and cell death was measured with LDH assay. Data shown as mean ± SEM of pooled data from three independent experiments. Statistical analyses were performed using a two-tailed *t* test. **(D, E)** RNH1-KO THP-1 cells transiently infected with GFP tagged control or RNH1 expressing lentivirus particles. **(D)** Cell lysates were analysed by Western blot to demonstrate the RNH1 reconstitution in RNH1-KO THP1 cells. Blots are representative of three independent experiments. **(E)** These cells were stimulated with Nigericin (5 μM) for 1 h or with MSU (500 μg) or Gramicidin A (30 μg) for 5 h. Supernatants were collected and IL-1β ELISA was performed. Data shown as mean ± SEM of pooled data from three independent experiments. **(F, G)** Total cell lysates from THP1 cells constitutively expressing control or Flag-RNH1 were analysed by Western blot with indicated antibodies to demonstrate RNH1 overexpression (F). Blots are representative of three independent experiments. These cells were stimulated with Nigericin (5 μM) for 1 h or with MSU (500 μg) or Gramicidin A (30 μg) for 5 h. **(G)** Supernatants were collected and IL-1β ELISA was performed (G). Data shown as mean ± SEM of pooled data from three independent experiments. **(H, I)** Immunofluorescence microscopy analysis of ASC specks in THP1 cells stimulated with Nigericin (5 μM) for 1 h. DNA staining is shown in blue (DAPI) and ASC staining is shown in red. Arrowheads indicate ASC inflammasome specks. (Scale bar: 100 μm) (H). Quantification of ASC specks (I). Data shown as mean ± SEM of pooled data analyzing at least 10 fields from three independent experiments. Source data are available for this figure.

**Figure S1.**
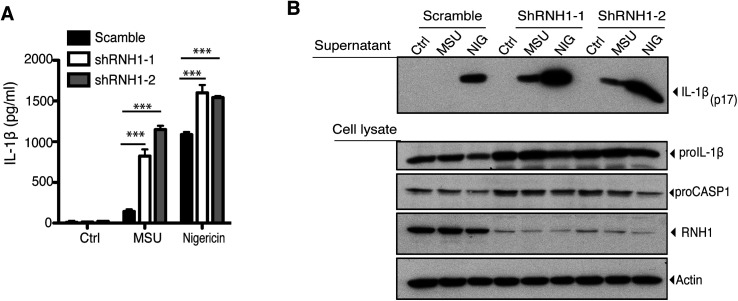
RNH1 knockdown aggravated NLRP3 inflammasome activation. **(A, B)** PMA differentiated scramble and RNH1 knockdown THP1 cells were stimulated with Nigericin (NIG) (5 μM) for 1 h and for 5 h with MSU (500 μg). **(A)** Supernatant was collected and IL-1β ELISA was performed. Data are means ± SEM of pooled data from three independent experiments. Statistical analyses were performed using a two-tailed *t* test. **(B)** Cell lysates and supernatants were analysed for pro- and cleaved-forms of IL-1β by Western blot. Blots were representative of two independent experiments. Source data are available for this figure.

**Figure S2.**
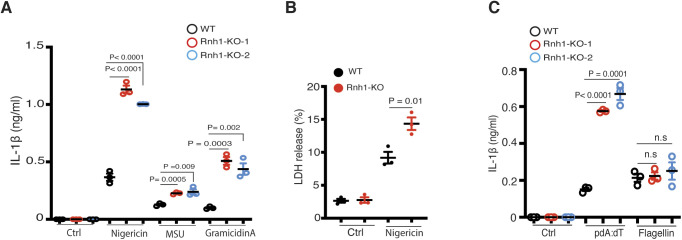
Loss of RNH1 increased inflammasome activation in iMAC cells. **(A)** WT and Rnh1-KO iMAC cells were primed with 500 ng of LPS for 3 h and then stimulated with Nigericin (5 μM) for 1 h or with MSU (500 μg) and Gramicidin A (30 μg) for 5 h. Supernatant were collected and IL-1β ELISA was performed. **(B)** WT and Rnh1-KO iMAC cells were primed with 500 ng of LPS for 3 h and then stimulated with Nigericin (5 μM) for 8 h. Supernatants were collected, and cell death was measured by LDH assay. **(C)** iMAC cells were primed with 500 ng of LPS for 3 h and then transfected with AIM2 agonist poly dA:dT (5 μg) or NAIP/NLRC4 agonist cytosolic flagellin (600 ng) for 5 h. Supernatants were collected and IL-1β ELISA was performed. All the data are means ± SEM of pooled data from three independent experiments. Statistical analyses were performed using a two-tailed *t* test. Source data are available for this figure.

NLRP3 is the most studied inflammasome and is activated by several pathogens and danger stimuli (Broz & Dixit, 2016). To investigate the role of RNH1 in NLRP3 inflammasome activation, we generated RNH1-deficient (RNH1-KO) THP1 cells using the CRISPR/cas9 system and stimulated them with NLRP3 agonists. RNH1-KO THP1 cells showed increased mature IL-1β production and caspase-1 cleavage compared with wild-type cells (WT) (Fig 2A and B). In addition, we also found increased pro-IL-1β and pro-caspase-1 in unstimulated RNH1-KO cells compared with WT (Fig 2B). shRNA knockdown studies in THP1 cells also showed similar results (Fig S1A and B). Because human and mouse RNH1 share 72.8% homology at the protein level, we also checked NLRP3 activation in mouse-derived immortalized macrophages (iMAC cells) (Broz et al, 2010). RNH1-KO iMAC cells also showed increased IL-1β production in response to NLRP3 agonists (Fig S2A). These data suggest that RNH1 limits NLRP3 inflammasome activation in both human and mouse cells. Inflammasome activation also triggers pyroptosis, a type of inflammatory cell death (Miao et al, 2011). We indeed observed an increased trend of cell death in RNH1-KO cells compared with WT cells upon NLRP3 activation (Figs 2C and S2B). To confirm the inhibitory function of RNH1 on inflammasome activation, we performed a transient reconstitution of RNH1 in RNH1-KO THP1 cells using lentiviral transduction, followed by stimulation with NLRP3 agonists. Reconstitution of RNH1 caused a decrease in IL-1β secretion (Fig 2D and E). We also generated stable RNH1 overexpressing THP1 cells and stimulated them with NLRP3 agonists. RNH1 overexpression decreased IL-1β secretion, thereby confirming that RNH1 negatively regulates NLRP3 inflammasome activation (Fig 2F and G). Furthermore, RNH1-KO THP1 cells also showed enhanced ASC speck formation upon Nigericin stimulation, which is a direct indicator of inflammasome activation (Stutz et al, 2013) (Fig 2H and I). Taken together, these results suggest that RNH1 dampens NLRP3 inflammasome activation.

### RNH1 regulates NF-kB activation and is involved in the priming of NLRP3

**Figure 3.**
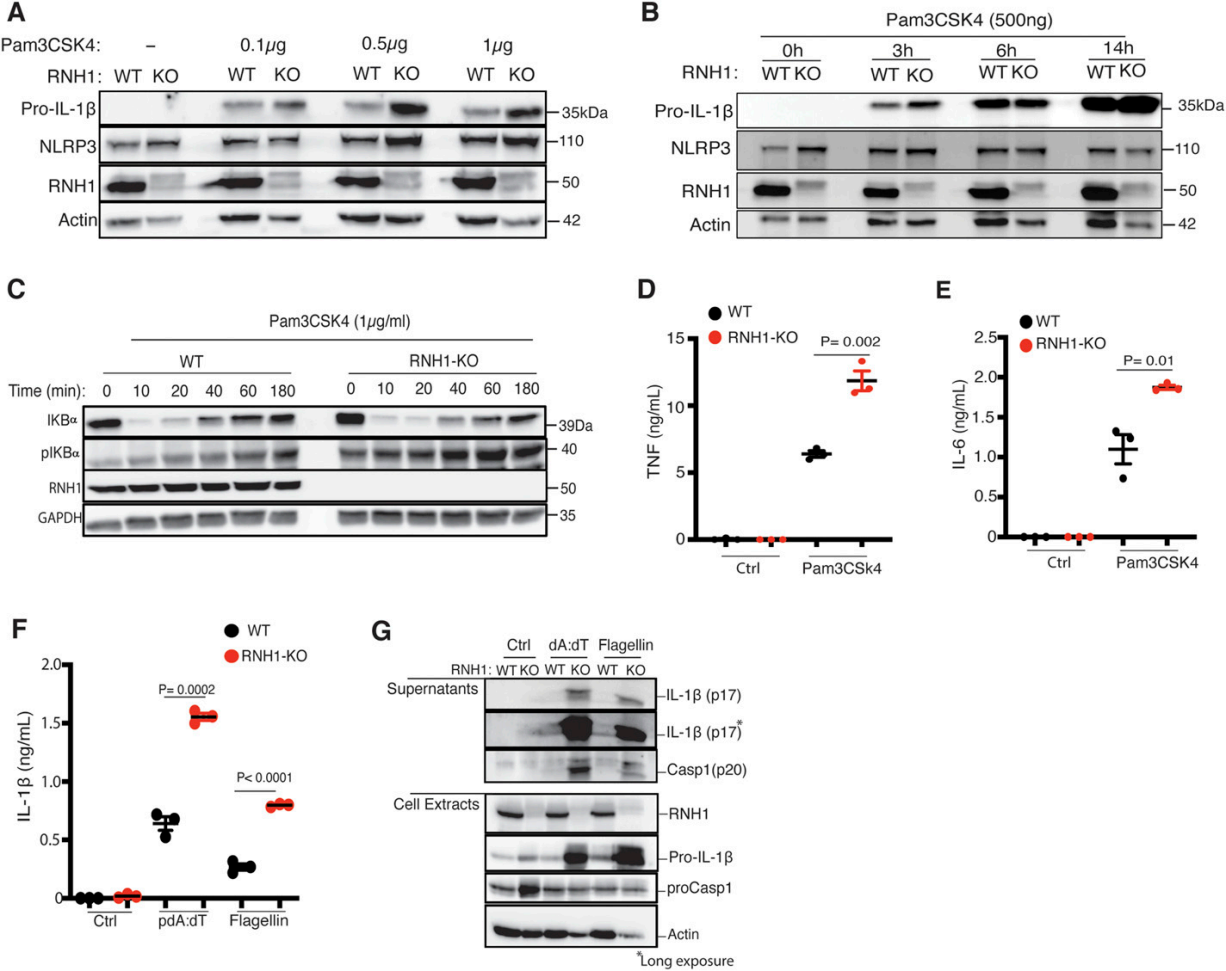
RNH1 negatively regulates activation of NF-κB, AIM2 and NAIP/NLRC4. **(A, B)** Undifferentiated WT and RNH1-KO THP1 cells were stimulated with TLR2 ligand Pam3CSK4 in a dose- (A) and time- (B) dependent manner as indicated. Total cell lysates analysed for pro-IL-1β and NLRP3 expression by Western blot. Blots are representative of three independent experiments. **(C)** Undifferentiated WT and RNH1-KO THP1 cells were stimulated with Pam3CSK4 (1 μg/ml) for different time points as indicated. Total cell lysates analysed for NF-κB activation by Western blot with indicated antibodies. Blots are representative of three independent experiments. **(D, E)** Undifferentiated WT and RNH1-KO THP1 cells were stimulated with Pam3CSK4 (1 μg/ml) for 6 and 18 h. Supernatants were analysed for TNF and IL-6 by ELISA, respectively. Data shown as mean ± SEM of pooled data from three independent experiments. **(F, G)** PMA differentiated WT and RNH1-KO THP1 cells were transfected with AIM2 agonist poly dA:dT (5 μg) or NAIP/NLRC4 agonist cytosolic flagellin (600 ng) for 5 h. **(F)** Supernatants were collected and IL-1β ELISA was performed (F). Data shown as mean ± SEM of pooled data from three independent experiments. Statistical analyses were performed using a two-tailed *t* test. **(G)** Cell lysates and supernatants were analysed for pro- and cleaved-forms of caspase-1 and IL-1β by Western blot (G). Blots are representative of three independent experiments. Source data are available for this figure.

Activation of the canonical NLRP3 inflammasome occurs in two steps (Latz et al, 2013). The priming step requires activation of nuclear factor kappa B (NF-κB), for example, when LPS binds to TLR4, which subsequently up-regulates NLRP3 and pro-IL-1β via activation of NF-κB. The second step requires a signal initiated by pathogen-associated molecular patterns or danger-associated molecular patterns, which leads to NLRP3 activation and formation of an inflammasome complex with ASC and caspase-1. In RNH1-KO cells, we found increased levels of pro-IL-1β suggesting that RNH1 may also be involved in reducing the priming signal (Fig 2B). To further understand this, WT and RNH1-KO THP1 cells were stimulated with the TLR2 ligand Pam3CSK4. RNH1-KO cells showed increased pro-IL-1β and NLRP3 expression in both a time- and Pam3CSK4 concentration–dependent manner, supporting the involvement of RNH1 in priming (Fig 3A and B). The nuclear translocation and activity of NF-κB is tightly controlled by IκBα—the inhibitor of NF-κB. Phosphorylation of IκBα triggers its degradation and NF-κB activation. Interestingly, RNH1-KO cells also displayed decreased levels of IκBα and increased levels of phospho-IκBα compared with WT cells (Fig 3C). To further confirm NF-κB activation, we monitored the production of pro-inflammatory cytokines and found that RNH1-KO cells exhibited enhanced secretion of TNF and IL-6 compared with WT cells (Fig 3D and E). Collectively, these results suggest that RNH1 decreases NF-κB activation and therefore reduces the priming step.

### RNH1 deficiency increases AIM2 and NAIP/NLRC4 inflammasome activation

**Figure S3.**
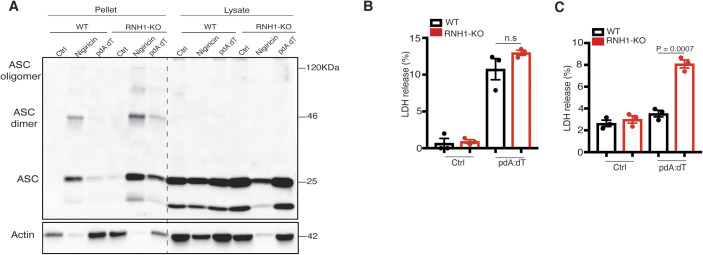
Loss of RNH1 increased ASC oligomerization. **(A)** PMA-differentiated WT and RNH1-KO THP1 were treated with Nigericin (5 μM) for 1 h and poly(dA:dT) (5 μg) for 5 h. Cross-linked pellets (Pellets) or soluble lysates (Lysates) were immunoblotted for ASC. Blots are representative of three independent experiments. **(B, C)** WT and RNH1-KO THP1 and iMAC cells were stimulated with AIM2 agonist poly dA:dT (5 μg) for 18 h. Supernatant were collected and cell death was measured by LDH assay. Data are means ± SEM of pooled data from three independent experiments. Statistical analyses were performed using a two-tailed *t* test. Source data are available for this figure.

To check whether RNH1 is also involved in activation of AIM2 and NAIP/NLRC4 inflammasomes, we stimulated WT and RNH1-KO THP1 cells with their agonists. We used cytosolic dsDNA (pdA:dT) and flagellin to activate AIM2 and NAIP/NLRC4 inflammasomes, respectively. Interestingly, RNH1-KO showed an increase in mature IL-1β production and caspase-1 cleavage compared with WT cells (Fig 3F and G), suggesting increased AIM2 and NAIP/NLRC4 inflammasome activation in RNH1-KO THP1 cells. We also found increased AIM2 inflammasome activation in mouse iMAC cells; however, there was no significant increase in cytosolic flagellin-induced NAIP/NLRC4 inflammasome activation (Fig S2C). Further studies are required to understand cell-type and species-specific differences in RNH1-mediated flagellin-induced inflammasome activation. To further confirm inflammasome activation, we checked ASC oligomerization by monitoring the abundance of ASC in the insoluble cell fraction (Lugrin & Martinon, 2017). As expected, the loss of RNH1 increased ASC oligomerization in response to NLRP3 and AIM2 inflammasome activators (Fig S3A). We also found an increased trend of cell death in RNH1-KO cells by AIM2 activation (Fig S3B and C). Together, these results suggest that RNH1 also negatively regulates AIM2 and NAIP/NLRC4 inflammasome activation.

### RNH1 mediates degradation of caspase-1 through the proteasome

**Figure 4.**
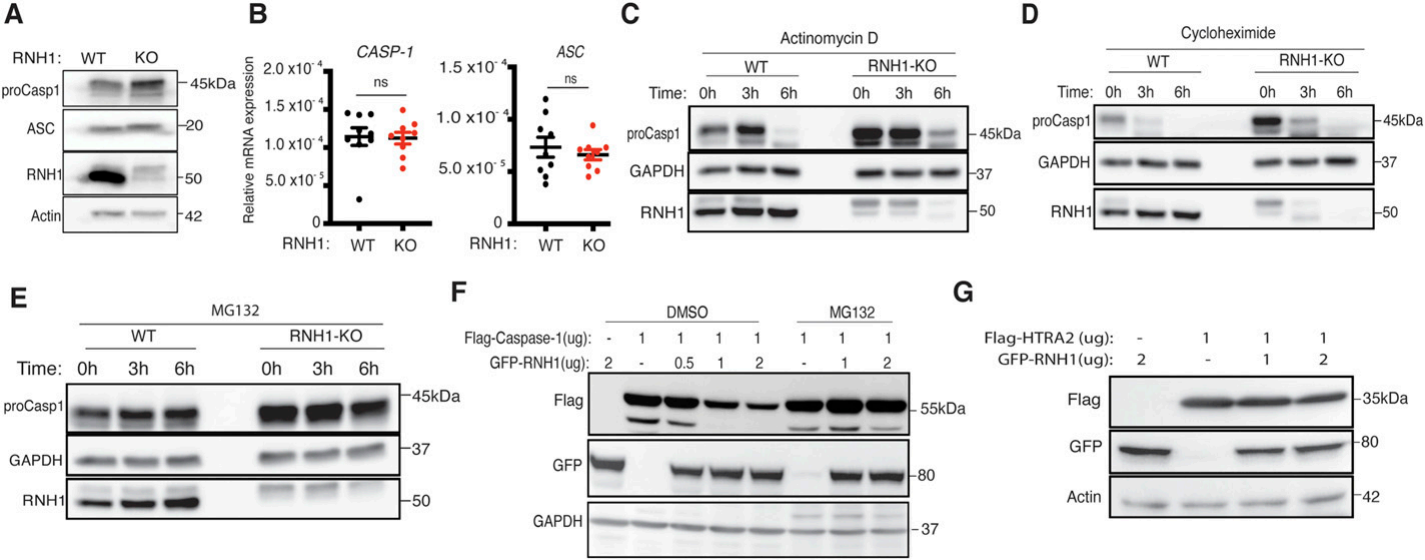
RNH1 increases caspase-1 degradation through the proteasome. **(A)** Total cell lysates from WT and RNH1-KO THP1 cells were analysed for pro-caspase-1 and ASC expression by Western blot. Blots are representative of two independent experiments. **(B)** qRT-PCR analysis for *CASP-1* and *ASC* mRNAs from WT and RNH1-KO THP1 cells. mRNA levels are normalized to 18S rRNA expression. Data shown as mean ± SEM from three independent experiments. **(C, D, E)** WT and RNH1-KO THP1 cells were treated with actinomycin D or cycloheximide or with the proteasome inhibitor MG-132 for indicated time duration. Cell lysates were analysed for pro-caspase-1 protein levels by Western blot. Blots are representative of three independent experiments. **(F)** HEK293T cells were treated with or without MG-132 and transfected with Flag-tagged caspase-1 with different concentration of GFP-tagged RNH1 plasmid as indicated. Cells were harvested and cell lysates were analysed for caspase-1 by Western blot with the indicated antibodies. Blots are representative of three independent experiments. **(G)** HEK293T cells were transfected with Flag-tagged OMI/HTRA2 plasmid with different concentrations of GFP-tagged RNH1 plasmid as indicated. Cells were harvested and cell lysates were analysed by Western blot with indicated antibodies. Blots are representative of three independent experiments. CASP-1 (caspase-1), ASC (apoptosis-associated speck-like protein containing a caspase activation and recruitment domain), HTRA2 (High temperature requirement protein A2), and GFP (green fluorescent protein). Source data are available for this figure.

The above results suggest that RNH1 inhibits not only NLRP3 inflammasome but also AIM2 and NAIP/NLRC4 – possibly by acting on common downstream effector/s. Interestingly, pro-caspase-1 protein levels were increased in RNH1-KO cells at a steady state (Figs 2B and 4A). Recently, it has been reported that caspase-1 levels are regulated at the transcriptional level by NF-κB activation (Lee et al, 2015). However, qPCR analysis showed that expression of both *caspase-1* and *ASC* are equivalent in WT and RNH1 KO cells (Fig 4B), indicating that RNH1 does not regulate caspase-1 at the transcriptional level. To check whether RNH1 regulates caspase-1 expression at the translational or post-translational level, we performed chase experiments using actinomycin-D, cycloheximide (CHX), and the proteasome inhibitor MG-132 in WT and RNH1-KO THP1 cells. Actinomycin-D and CHX inhibit transcription and translation, respectively. Actinomycin-D and CHX treatment decreased caspase-1 protein levels in both WT and RNH1-KO cells with similar kinetics (Fig 4C and D). Interestingly, proteasome blockade with MG-132 increased caspase-1 protein levels in WT cells. However, in RNH1-KO cells, caspase-1 levels were not increased over time with MG-132 treatment. This suggests that loss of RNH1 might stabilize caspase-1 protein by inhibiting proteasome mediated degradation (Fig 4E). To test this further, we expressed full-length Flag-caspase-1 in the presence or absence of GFP-RNH1 in HEK293T cells. Strikingly, caspase-1 protein levels decreased with increasing concentration of RNH1, an effect that was blocked in the presence of MG-132 (Fig 4F). It has been reported that overexpression of caspase-1 induces self-cleavage (Li et al, 2008); however, we did not observe caspase-1 self-cleavage at the concentration that we used in our experiments. Interestingly, we did not observe a similar decrease in another protease, OMI/HTRA2 (High temperature requirement protein A2) (Walle et al, 2008) (Fig 4G). This implies that RNH1 might specifically mediate proteasomal degradation of caspase-1 or of a defined set of proteins. Collectively, these results suggest that RNH1 increases proteasome-mediated caspase-1 degradation. However, further studies are required to understand the molecular mechanism of RNH1-mediated caspase-1 degradation.

### Loss of RNH1 in mice increases inflammation and lethality upon inflammasome activation

**Figure 5.**
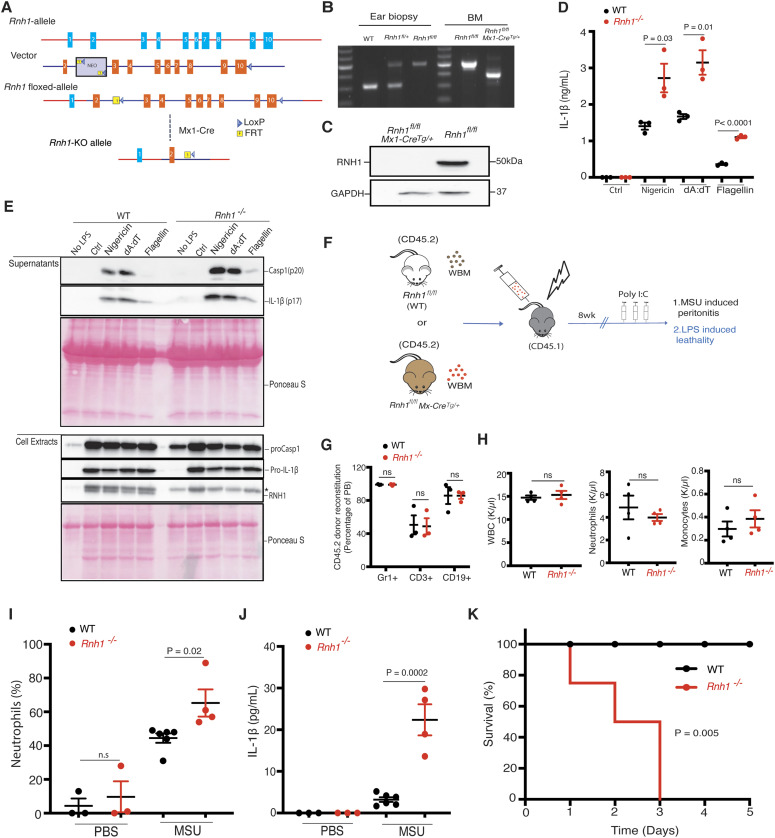
*Rnh1* deficiency promotes inflammation in mice. **(A)** Schematic showing design of *Rnh1-*floxed targeting vector (see the Materials and Methods section for details). **(B)** DNA isolated from mouse ear biopsies and BM was genotyped by PCR. Primers were designed to distinguish WT and floxed allele sequence. 314 bp size corresponds to WT, and 514 bp size corresponds to floxed gene. To detect *Rnh1* deletion after Cre-recombination in *Rnh1*^*fl/fl*^ MxCre^+^ mice (after 1 wk of polyIC treatment), a third primer was used which amplifies 365 bp size (see the Materials and Methods section). **(C)** Total protein lysates from BM cells of WT and *Rnh1*^*−/−*^ mice were analysed by Western blot with the indicated antibodies. Blots are representative of three independent experiments. **(D, E)** BMDMs from WT and *Rnh1*^*−/−*^ mice primed with LPS (100 ng) for 3 h and then cells were stimulated with Nigericin (5 μM) for 1 h or transfected with poly dA:dT (5 μg) or flagellin (600 ng) for 5 h. **(D)** Supernatants were collected and IL-1β ELISA was performed. Data shown as mean ± SEM and representative of three independent experiments. Statistical analyses were performed using a two-tailed *t* test. **(E)** Cell lysates and supernatants were analysed for pro- and cleaved-forms of caspase-1 and IL-1β by Western blot. Blots were representative of two independent experiments. * non-specific band from previous antibody incubation. **(F)** Schematic showing in vivo experimental setup. BM was transplanted from WT (*Rnh1*^*fl/fl*^) or *Rnh1*^*−/−*^ *(Rnh1*^*fl/fl*^ MxCre^+^) (CD45.2) into irradiated recipients (CD45.1). After 8 wk of reconstitution mice were treated with Poly(I:C) (300 μg/mouse) once every 2 d for three doses. 4 d later mice were used for the MSU peritonitis and LPS endotoxemia lethality models. **(G)** Post transplantation reconstitution levels were monitored after 8 wk in the peripheral blood (PB) as indicated (n = 3 mice). Data shown as mean ± SEM. *P*-values were determined by a two-tailed *t* test. **(H)** PB counts of WBC, neutrophils, and monocytes in WT and *Rnh1*^*−/−*^ mice 1 wk after polyIC injections. Data are shown are mean ± SEM. *P*-values were determined by a two-tailed *t* test (n = 3). **(I, J)** WT and *Rnh1*^*−/−*^ mice received IP injection of MSU (1 mg/mouse) or sterile PBS. **(I)** After 12 h, peritoneal lavage fluid was taken, and neutrophils infiltration analysed by FACS (I). **(J)** Peritoneal lavage supernatants were collected and IL-1β levels were quantified by ELISA (J). Data shown as mean ± SEM and *P*-values were determined by a two-tailed *t* test (n = 3–6 per group). **(K)** Kaplan–Meier survival curves of WT and *Rnh1*^*−/−*^ mice after LPS (10 mg/kg) treatment (n = 4 mice). Survival curves were tested with log-rank (Mantel–Cox) test (*P* = 0.005). Source data are available for this figure.

Constitutive deletion of *Rnh1* leads to embryonic lethality in mice (Chennupati et al, 2018). We generated *Rnh1* conditional knockout mice (*Rnh1*^*fl/fl*^) to understand the in vivo relevance of our results (Fig 5A). *Rnh1*^*fl/fl*^ mice were crossed with transgenic interferon-inducible *Mx1-Cre* mice to generate an inducible *Rnh1*^*−/−*^ mouse model (*Rnh1* ^*fl/f;Mx1-Tg+*^). Administration of polyinosinic:polycytidylic acid (polyI:C) into *Rnh1* ^*fl/f;Mx1-Tg+*^ mice leads to cre-recombination and complete deletion of RNH1 protein expression in hematopoietic organs such as the BM, which we refer to as *Rnh1*^*−/−*^ mice (Fig 5B and C). We generated BMDMs from WT (*Rnh1*^*fl/fl*^) and *Rnh1*^*−/−*^ mice and then stimulated them with inflammasome activators. As observed in cell lines, loss of RNH1 in primary mouse BMDMs led to increased mature IL-1β production and caspase-1 cleavage compared with WT cells upon stimulation with NLRP3, AIM2, and NAIP/NLRC4 agonists (Fig 5D and E). To investigate the in vivo role of RNH1 in inflammasome activation, we performed mouse models of caspase-1–dependent MSU-induced peritonitis and LPS-induced lethality (Li et al, 1995; Martinon et al, 2006; Man et al, 2017) (Fig 5F). Because *Mx1-Cre* activation also deletes genes in non-haematopoietic organs (Kühn et al, 1995), we transplanted WT (*Rnh1*^*fl/fl*^) and *Rnh1*^*−/−*^ (*Rnh1*^*fl/fl*^ *Mx1-Cre*^*+*^) BM into irradiated CD45.1 congenic mice to exclude *Rnh1* deletion effects from non-haematopoietic cells. After 8 wk, we checked reconstitution levels in WT and *Rnh1*^*−/−*^ BM transplanted CD45.1 congenic mice. We found comparable reconstitution in WT and *Rnh1*^*−/−*^ BM transplanted CD45.1 congenic mice via FACS analysis (Fig 5G). *Rnh1* was deleted by giving three rounds of 200 μg of polyI:C injections. 4 d after the last injection, mice were analysed for peripheral blood parameters. At this time point, we observed no major difference in leukocyte numbers between WT and *Rnh1*^*−/−*^ BM transplanted CD45.1 congenic mice (Fig 5H). In a MSU-induced model of peritonitis, *Rnh1*^*−/−*^ mice showed increased neutrophil infiltration in the peritoneal cavity compared with WT mice (Fig 5I). Consistent with this, we also found increased IL-1β production in the peritoneal lavage of *Rnh1*^*−/−*^ mice compared with WT mice (Fig 5J). We next challenged mice with LPS at 10 mg/kg. This dose was lethal to *Rnh1*^*−/−*^ mice, but all WT mice survived (Fig 5K). Altogether, these results demonstrate that *Rnh1* deficiency promotes excessive inflammasome activation and increases inflammation in vivo.

### RNH1 is negatively associated with disease severity in COVID-19 patients

**Figure 6.**
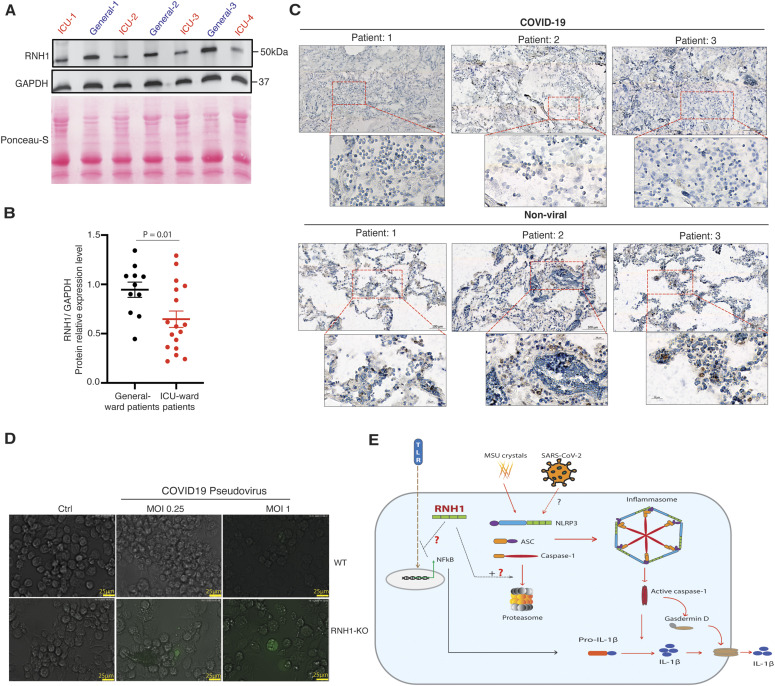
Decreased RNH1 expression associates with disease severity in COVID-19. **(A)** Total cell lysates from peripheral blood buffy coats of intensive care unit and general ward admitted COVID-19 patients were analysed for RNH1 protein levels by Western blot. Blots were repeated three times. **(B)** RNH1 protein levels from blots were quantified by ImageJ analysis for each patient and normalized with respective GAPDH protein levels. Data shown as mean ± SEM and *P*-values were determined by a two-tailed *t* test. **(C)** Postmortem lung tissue from deceased persons with either COVID-19 or non-viral causes of death were stained for RNH1 and imaged using a Zeiss axioscan Z1. Subsequent image analysis was performed using Zeiss ZEN software to extract images at different magnifications. Insets show higher magnification of area indicated in the red boxes. Brown staining indicates RNH1 positive cells. **(D)** PMA differentiated WT and RNH1-KO THP1 cells were infected with SARS-CoV-2 pseudovirus for 24 h. Infection efficiency was monitored by measuring GFP signal with fluorescence microscopy. Images are representative of two independent experiments. (Scale bar 25 μm). **(E)** Illustration of RNH1-mediated anti-inflammatory mechanisms. First, RNH1 could potentially inhibit NF-κB signaling through an unknown mechanism. Second, RNH1 regulates inflammasome activation by controlling caspase-1 protein levels via proteasome-mediated degradation. Source data are available for this figure.

**Figure S4.**
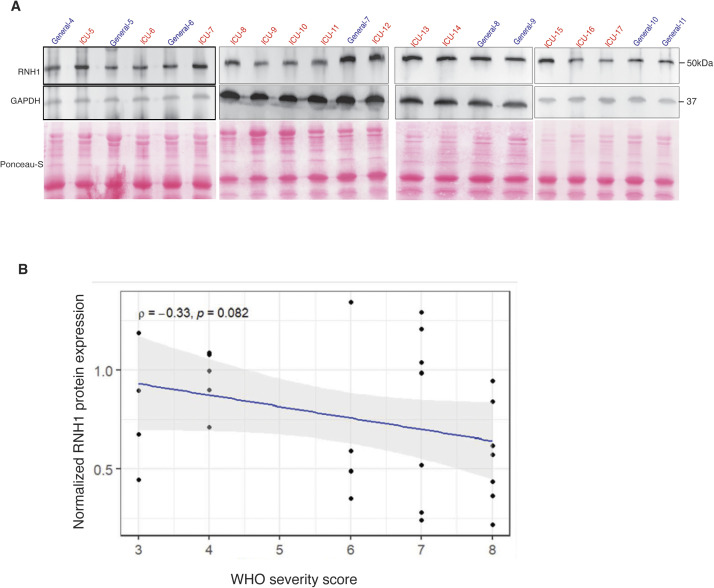
RNH1 expression negatively associates with disease severity in COVID-19 patients. **(A)** Total cell lysates from peripheral blood buffy coats of intensive care unit and general ward admitted COVID-19 patients were analysed for RNH1 protein levels by Western blot. Blots were repeated for three times. **(B)** Spearman’s rank correlation statistical test was performed using each individual patient’s normalized RNH1 protein levels with World Health Organization severity scores. (Coefficient ρ [rho] = −0.33; *P* = 0.0824). Source data are available for this figure.

**Figure S5.**
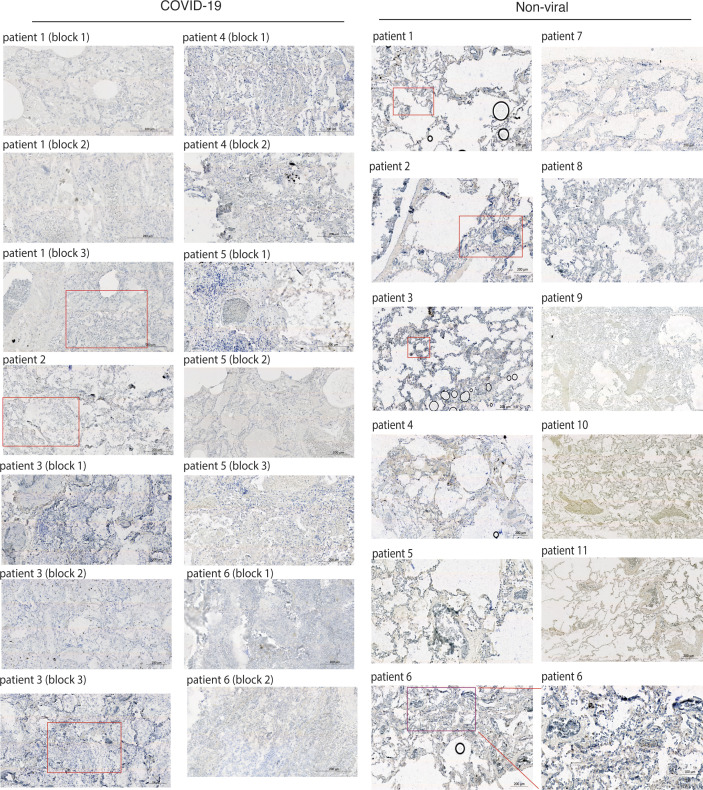
RNH1 expression is decreased in COVID-19 patients lung biopsies compared with non-viral patients. Postmortem lung tissue from deceased persons with either COVID-19 (n = 8) or non-viral (n = 13) causes of death were stained for RNH1 and imaged using a Zeiss AxioScan Z1. Subsequent image analysis was performed using Zeiss ZEN software to extract images at different magnifications. Insets show higher magnification of area indicated in the red boxes. Brown staining indicates RNH1-positive cells. Source data are available for this figure.

Recent studies suggest that pronounced inflammation and inflammasome activation in patients with COVID-19 correlates with increased disease severity and poor prognosis (van den Berg & Velde, 2020; Mehta et al, 2020; Jose & Manuel, 2020; Toldo et al, 2020; Rodrigues et al, 2021; Kroemer et al, 2020; Ferreira et al, 2021). Because our results suggest that RNH1 negatively regulates NFκB and inflammasome activation, we investigated RNH1 levels in COVID-19 patients. Buffy coat samples from critically ill COVID-19 patients admitted to the intensive care unit (ICU) (n = 17) and COVID-19 patients admitted to general COVID-19 ward (n = 11 patients) were analysed for RNH1 protein expression by Western blot (see the Materials and Methods section for details). Disease severity of these patients was reported with WHO severity scores and comorbidity data and routine clinical parameters showed differences between general ward and ICU patients (Table 1). Strikingly, COVID-19 patients in ICU had less RNH1 expression when compared with COVID-19 patients in general ward (RNH1 mean difference: 0.299; 95% CI : 0.052–0.547, effect size: large [d: 0.961]) (Figs 6A and B and S4A). The decreased RNH1 expression in patients admitted to ICU is unlikely to reflect leukocyte numbers because leukocyte numbers were rather increased in ICU patients compared with general ward COVID-19 patients (Table 1). We performed Spearman’s rank correlation statistical test using each individual patient’s RNH1 protein levels with WHO severity scores. Although there is no significant correlation for RNH1 expression and COVID-19 severity, a negative trend is observed between these two variables (Fig S4B). To further support these findings, we analysed RNH1 expression in lung sections of patients from an independent study in the UK who deceased either from COVID-19 (n = 8) or from non-viral causes (n = 13) (Table S1). In line with the data above, RNH1 expression is largely absent in the lungs of deceased COVID-19 patients, whereas patients who succumbed to non-viral causes did show RNH1 staining in infiltrating cells in the lung (Figs 6C and S5). This data illustrates a loss of RNH1 expression in immune cells during severe COVID-19, which may suggest a crucial role for RNH1 in controlling severe inflammation.

Table S1 COVID-19 demographic data from independent study in the UK.

**Table 1.** Baseline demographics, disease severity, and clinical outcome.

		ICU patients with COVID-19	Normal ward COVID-19 patients	Total cohort (n = 28)	Between group
		(n = 17)	(n = 11)		*P*-value
Demographics	Age (years)	69.6 (66.9, 77.2)	55 (49, 70.5)	68.8 (54.95, 73.33)	0.082
	Gender (male, %)	14 (82.4)	9 (81.8)	23 (82.1)	1
	Body mass index	26.2 (25.25, 29.98)	27.3 (24.44, 29.22)	26.6 (24.89, 29.79)	0.957
	APACHE-II score (first 24 h)	21 (19, 26)	—	—	—
	SAPS II score (first 24 h)	45 (42, 59)	—	—	—
	SOFA score (baseline)	8 (7, 10)	—	—	—
Comorbidity data	Charlson comorbidity index	4 (3, 7)	2 (0.5, 3.5)	4 (2, 5.5)	0.012
	Myocardial infarction (No./%)	4 (24%)	0 (0%)	4 (14%)	0.13
	Chronic heart failure (No./%)	1 (5.9%)	0 (0%)	1 (3.6%)	>0.9
	Peripheral vascular disease (No./%)	1 (5.9%)	0 (0%)	1 (3.6%)	>0.9
	Cerebrovascular accident (No./%)	2 (12%)	1 (9.1%)	3 (11%)	>0.9
	Dementia (No./%)	0 (0%)	1 (9.1%)	1 (3.6%)	0.4
	COPD (No./%)	2 (12%)	1 (9.1%)	3 (11%)	>0.9
	Connective tissue disease (No./%)	0 (0%)	0 (0%)	0 (0%)	—
	Peptic ulcer disease (No./%)	0 (0%)	0 (0%)	0 (0%)	—
	Liver disease (No./%)	2 (12%)	1 (9.1%)	3 (11%)	>0.9
	Diabetes (No./%)	7 (41%)	2 (18%)	9 (32%)	0.2
	Hemiplegia (No./%)	0 (0%)	0 (0%)	0 (0%)	—
	Moderate to severe CKD (No./%)	2 (12%)	0 (0%)	2 (7.1%)	0.5
	Solid tumor (No./%)	1 (5.9%)	0 (0%)	1 (3.6%)	>0.9
	Leukemia (No./%)	0 (0%)	0 (0%)	0 (0%)	—
	Lymphoma (No./%)	1 (5.9%)	0 (0%)	1 (3.6%)	>0.9
	HIV/AIDS (No./%)	1 (5.9%)	0 (0%)	1 (3.6%)	>0.9
Laboratory data	C-reactive protein (mg/l)	148 (87, 315)	43 (12.5, 101.5)	100.5 (54.25, 196.5)	0.001
	Procalcitonin levels (ng/ml)	1.1 (0.35, 9.82)	0.2 (0.15, 0.24)	0.4 (0.25, 5)	0.004
	Total leukocyte count (G/l)	8.4 (6.98, 10.5)	6.1 (4.82, 6.88)	7 (5.95, 9.94)	0.004
	Total lymphocyte count (G/l)	0.6 (0.44, 0.77)	1.1 (0.88, 1.36)	0.7 (0.51, 1.12)	0.1
	Platelet count (G/l)	208 (117, 225)	203 (153, 247)	205.5 (147.25, 231.25)	0.572
	Serum potassium (mmol/l)	4.2 (4, 4.6)	3.8 (3.7, 3.9)	4 (3.9, 4.2)	0
	Serum creatinine (μmol/l)	103 (86, 160)	73 (57.5, 86.5)	89 (68, 132.25)	0.008
	D-Dimers (μg/l)	1,291 (1,183, 1,447)	490 (459, 1,276)	1,262 ((1,017, 1,479.5)	0.482
	PaO2/FiO2	87 (68, 128)	309 (290, 312)	161 (86, 295)	<0.001
Maximal modality of ventilation (during all hospitalisation stay)	No supplementary oxygen required	0 (0%)	6 (54.5%)	6 (21.4%)	< 0.001
	Low-flow nasal cannula	0 (0%)	4 (36.4%)	4 (14.3%)	< 0.001
	Simple face mask	0 (0%)	0 (0%)	0 (0%)	—
	Non-rebreather mask	0 (0%)	0 (0%)	0 (0%)	—
	High-flow nasal cannula	0 (0%)	0 (0%)	0 (0%)	—
	Non-invasive mechanical ventilation	0 (0%)	0 (0%)	0 (0%)	—
	Invasive mechanical ventilation	17 (100%)	1 (9.1%)^a^	18 (64.3%)^a^	< 0.001
Follow-up	World Health Organization score	7 (7, 8)	4 (3, 4)	6.5 (4, 7.25)	< 0.001
	Days on ICU	14 (8, 21)	—^a^	—	—
	Days in hospital	17 (9, 24)	6 (3.5, 8)	10.5 (5.75, 21.75)	0.004
	Days on antibiotics	10 (8, 14)	0.5 (0, 4)	8 (3, 14)	0.001
	Total days on mechanical ventilation	15 (9, 20.56)	—^a^	—	—
	Renal replacement at any time	9 (52.9)	—	—	—
	(No./%)				
	On vasopressors at any time	17 (100)	—^a^	—	—
	(No./%)				
	Norepinephrine dose	2.9 (1.9, 5.77)	—^a^	—	—
	(Cumulative dose/ICU days; mg)				
	ICU Mortality (No./%)	6 (35.3)	—	—	—
	Hospital mortality (No./%)	6 (35.3)	0 (0)	6 (21.4)	0.055

Demographical data, baseline comorbidities, laboratory data, and clinical follow-up are given for patients according to the initial admission (ICU versus normal ward). G = Giga, L = Liters, APACHE-II = Acute Physiology and Chronic Health Evaluation-II score, SAPS-2 = Simplified Acute Physiology Score-2, SOFA = sepsis-related organ failure assessment score, COPD = chronic obstructive pulmonary disease, HIV = human immunodeficiency virus, AIDS = acquired immunodeficiency syndrome. Numbers (No.) with percentages are given, as indicated. Continuous data are reported as median [quartiles]. Between-group *P*-values from Mann–Whitney U tests and Fisher’s exact tests are given for ICU versus non-ICU (normal ward) populations. Between group *P*-values are given for ICU versus non-ICU (normal ward) populations.

a Please note one patient with an initial normal ward admission has been later transferred to the ICU. The patient required mechanical ventilation and vasopressor treatment.

Recent studies document the critical role of monocytes and macrophages in SARS-CoV-2 infection (Desai et al, 2020; Ferreira et al, 2021). To check whether RNH1 affects the SARS-CoV-2 infection rate in macrophages, we infected WT and RNH1-KO macrophages differentiated from THP1 monocytes with SARS-CoV-2 pseudoviral particles (Hoffmann et al, 2020; Nie et al, 2020). These pseudoviral particles express SARS-CoV-2 spike protein together with eGFP as a reporter. Interestingly, we found increased eGFP+ cells in RNH1-KO cells compared with WT cells (Fig 6D). At a MOI of 0.25, we observed 1% of RNH1-KO cells were positive for eGFP. This increased to 2.5% at a MOI of 1; however, we did not find any eGFP+ cells in the WT at 0.25 MOI and very few (<0.5%) at 1 MOI. These results suggest that RNH1-KO cells are more susceptible to infection (Fig 6D). Collectively, our findings suggest that decreased expression of RNH1 associated with severity and increased inflammation in COVID-19 patients, and possibly increases the risk of SARS-CoV-2 infection.

## Discussion

Inflammasomes are multiprotein cytoplasmic complexes that induce potent inflammation and Gasdermin-D–mediated pyroptosis upon activation (Broz & Dixit, 2016). This process is critical for pathogen clearance and to maintain tissue homeostasis. However, uncontrolled inflammasome activation leads to excessive inflammation and tissue destruction, which is a primary cause of several inflammatory, autoimmune, and autoinflammatory diseases (Lamkanfi & Dixit, 2012; Strowig et al, 2012). Therefore, precise regulation of inflammasome signaling is necessary to prevent collateral damage while still preventing pathogen insurgence. Our results pointed to RNH1 as a new negative regulator of inflammasome activation. Conventionally, RNH1 is known to inhibit ribonucleases and protect RNA from degradation (Dickson et al, 2005). However, constitutive deletion of *Rnh1* in mice is embryonically lethal due to marked defects in erythropoiesis. These defects originate from decreased translation of the transcriptional erythropoiesis regulator GATA-1 rather than detectable effects on mRNA levels. This suggests that RNH1 fulfills functions beyond inhibition of ribonucleases (Chennupati et al, 2018). This is also supported by the present study in which RNH1 mainly exerts post-translational effects.

The RNH1 protein sequence consists of only LRRs and evolved via exon duplication (Haigis et al, 2002). Interestingly, RNH1 shares homology with the LRRs of NLRs, yet the function of RNH1 in NLR signaling is unknown. Although RNH1 is a ubiquitously expressed protein (Dickson et al, 2005), we demonstrate that it is highly expressed in myeloid cells and that its expression increases under inflammatory conditions, thus pointing to a potential role in regulating inflammation. Interestingly, we found that RNH1-deleted macrophages have increased IL-1β production, caspase-1 activation and pyroptosis in response to NLRP3 agonists, suggesting that RNH1 can potentially inhibit NLRP3-induced inflammation. Increased expression of RNH1 in inflammatory conditions may be a compensatory mechanism to dampen inflammation. In, we identified increased NF-κB signaling and increased expression of pro-IL-1β and NLRP3 in the absence of RNH1. This clearly indicates that RNH1 regulates NLRP3 inflammasome activation at the priming step as well as during the activating signal. We did not investigate MAPK pathways, which play a role in TLR induced inflammation (Arthur & Ley, 2013), and therefore cannot exclude that the MAPK pathway may mediate priming in RNH1-deleted cells. Previous reports suggest that reactive oxygen species (ROS) are involved both in priming and in NLRP3 inflammasome activation (Zhou et al, 2011). RNH1 protein contains numerous cysteine residues (e.g., 32 in human RNH1), whose sulfhydryl groups may play a structural role and have also been shown to protect against oxidative damage (Dickson et al, 2005). Thus, increased ROS production in the absence of RNH1 may be responsible for NLRP3 inflammasome activation. However, loss of RNH1 also increased AIM2 and NAIP/NLRC4 inflammasome activation, both of which do not require ROS. Therefore, these observations do not substantiate a major anti-ROS role of RNH1 to suppress activation of the NLRP3 inflammasome. Further studies are necessary to exclude the role of ROS in RNH1-mediated NLRP3 inflammasome regulation. From this study, RNH1 has emerged as an inhibitor of inflammasome activation. By suppressing inflammasome activation, RNH1 likely controls the extent of potentially dangerous immune activation.

In addition to increased canonical inflammasome activation and higher caspase-1 activity in RNH1-KO cells, we also observed increased caspase-1 protein levels in RNH1-KO cells compared with WT. Caspase-1 is a cysteine protease acting downstream of inflammasome activation. Upon activation, pro-caspase-1 is cleaved into two subunits (p10 and p20) and mediates proteolytic processing of pro-IL-1β and pro-IL-18 while promoting pyroptosis by cleaving gasdermin (Broz & Dixit, 2016). Few studies report mechanisms for the inhibition of caspase-1 function. Flightless–I binding can inhibit caspase-1 cleavage (Li et al, 2008; Man et al, 2017) and CARD only protein-1 (COP-1) has been shown to interact with the CARD domain of caspase-1 and inhibit its interaction with ASC (Druilhe et al, 2001; Pedraza-Alva et al, 2015). However, to our knowledge, regulation of caspase-1 expression at post-translational level as found in the present study has not been previously reported. Furthermore, we demonstrate that RNH1 promotes proteasome-mediated caspase-1 protein degradation, which was rescued by inhibiting proteasome function. Further investigation is required to understand how RNH1 regulates proteasome-mediated caspase-1 degradation. Caspase-1–deficient mice are resistant to LPS-induced lethality and MSU-induced peritonitis (Martinon et al, 2006; Man et al, 2017). Supporting a role for RNH1 in inflammasome activation and caspase-1 activity, *Rnh1*^*−/−*^ mice showed markedly increased susceptibility to LPS-induced lethality. Furthermore, *Rnh1*^*−/−*^ mice also displayed increased neutrophil infiltration in MSU-induced peritonitis. These results mirror observations obtained in vitro. Collectively, our study unravels a significant role of RNH1 in dampening inflammasome activation and demonstrates a previously unidentified regulation of caspase-1 through proteasome-mediated degradation (Fig 6E).

Uncontrolled inflammation mediates immune pathology in several inflammatory diseases, including the COVID-19 pandemic (Jose & Manuel, 2020; Mehta et al, 2020; Huang et al, 2020b). Recent studies have demonstrated a correlation of increased inflammasome activation and disease severity in COVID-19 patients (van den Berg & Velde, 2020; Toldo et al, 2020). We have shown that RNH1-KO THP1 cells are susceptible to infection with SARS-CoV-2 pseudovirus. Furthermore, we identified decreased RNH1 protein expression in COVID-19 patients with severe symptoms admitted to the ICU compared with COVID-19 patients with less severe symptoms admitted to the general ward, suggesting that RNH1 expression may be negatively related to the severity of the COVID-19 disease, and this was confirmed in an independent cohort of patients with COVID-19 using postmortem lung tissue. Although no statistically significant correlation was noted, this should be investigated in larger analyses. We showed that the expression of RNH1 protein is decreased in lung sections of patients with COVID-19 compared with patients who died from non-viral causes. This is contrary to what we have shown in systemic inflammatory conditions, where increased expression of RNH1 may be a compensatory mechanism to inhibit inflammation (Fig 1D). Therefore, it is unclear what drives the reduced expression of RNH1 in severe COVID-19 pathology. Understanding whether this is a common feature of severe inflammation, or of infection-driven inflammation, and what causes it is of interest.

### Limitations of the study

The data provided here using in vitro experiments and mouse models of inflammation support the role of RNH1 in suppressing inflammation. These experiments suggest that RNH1 is a new negative regulator of NLRP3 inflammasome activation and NFkb signaling. Because inflammasomes were involved in SARS-CoV-2 infection–mediated inflammation and pathology we also studied the role of RNH1 in COVID-19. We found a significant decrease in RNH1 protein expression in ICU COVID-19 patients compared with COVID-19 general ward patients. Furthermore, RNH1 expression is largely absent in the lungs of deceased patients with COVID-19 compared with patients who succumbed to non-viral causes. These results suggest a potential involvement of RNH1 in the severity of disease; however, there are limitations to the study. First, statistically, no significant correlation between the expression of the RNH1 protein and the WHO severity scores of COVID-19 patients. Further studies with a larger number of patient samples are required to clarify the correlation between RNH1 expression and disease severity in COVID-19 patients. Second, our studies have investigated the expression of RNH1 in COVID-19 patients at admission stage but not at other time points, so we do not know how the expression of RNH1 could affect the progression of the disease over time. More prospective studies by analyzing the level of the RNH1 protein at different intervals are necessary to understand the role of RNH1 in disease progression. Third, we cannot exclude an inflammation independent role of RNH1 in COVID-19 disease severity. Further molecular studies with RNH1 mouse models of COVID-19 infection are required to clarify this. Collectively, our studies introduce RNH1 as a new negative regulator of inflammasome activation. However, further studies are warranted to understand the molecular details of how RNH1 regulates the priming and activation signal of the NLRP3 inflammasome and how these mechanisms are dysregulated in human inflammatory diseases, including COVID-19 and sepsis.

## Materials and Methods

### Details of Rnh1 conditional-knockout mice generation and mouse experiments

*Rnh1* conditional-knockout (*Rnh1*^*fl/fl*^) mice were generated on a C57BL/6 background. A 13.8 kb region used to construct the targeting vector was first subcloned from a positively identified C57BL/6 BAC clone (RP23: 217N5) using a homologous recombination-based technique. The region was designed in a way that the long homology arm (LA) extends 6.23 kb 3′ to the single LoxP site. The short homology arm (SA) extends about 3.0 kb 5′ to the LoxP/FRT flanked Neo cassette. The single LoxP site is inserted downstream of exon 10, and the Neo cassette is inserted upstream of exon 3. The size of the target region is about 4.6 kb containing exons 3–10 (Fig 5A). *Rnh1* targeting vector was electroporated into (C57BL/6) embryonic stem (ES) cells. Targeted (C57BL/6 FLP) embryonic stem cells were microinjected into Balb/c blastocysts. Resulting chimeras with a high percentage black coat colour were mated to C57BL/6 WT mice to generate Neo deleted, F1 heterozygous offspring. Germline transmission was confirmed by PCR of tail genomic DNA. Screening of *Rnh1*^*fl/fl*^ mice by PCR genotyping was carried out using the following primers on ear genomic DNA: 5′-ACATGGTGTTCTGGG TGTACGGTGG-3′ (forward in the intron region after second exon), 5′-CTGAGTAAGGAC TGCTGGGCTGAG-3′ (reverse in the proximal *LoxP* region). This reaction amplifies 314 bp in size for WT allele and 514 bp size for floxed allele (Fig 5B). We crossed *Rnh1*^*fl/fl*^ mice with *Mx1*-*Cre* mouse strain (Jackson: 002527) to generate inducible mouse model (*Rnh1*^*fl/fl*^ *Mx1-Cre*^*+*^). To exclude non-haematopoietic deletion, total BM was isolated from WT (*Rnh1*^*fl/fl*^) or *Rnh1*^*fl/fl*^ *Mx1-Cre*^*+*^ mice and transplanted (7 × 10^6^ BM cells) into lethally irradiated CD45.1^+^ congenic recipient mice (8 wk old male mice) (B6.SJL-PtprcaPepcb/BoyCrl, from Charles River). After 8 wk of reconstitution, *Rnh1* was excised by giving three rounds of 200 μg poly(I:C) (Invivogen) using IP injections once in 2 d. We used these mice for MSU induced peritonitis model and LPS lethality model. To detect *Rnh1* deletion after Cre-recombination, we used a third primer 5′-AAGACCCATCCAGAGCCGAGG-3′ (reverse in the distal intron region) in above mentioned PCR reaction using DNA form BM cells, which amplifies 365 bp size (Fig 5B). Western blot was also performed in BM cells to check RNH1 protein expression (Fig 5C). Mouse peripheral blood was taken from the lateral tail vein with an EDTA coated Microvette 100 K3E (20.1278; Sarstedt). Full blood counts were measured on an IDEXX ProCyte Dx Hematology Analyzer (IDEXX Laboratories). The genotypes of the mice could not be blinded or randomized because of the experimental design.

All groups of mice (experimental and control mice) were age- and sex-matched. We used littermates for our experiments. All the cages were placed at the same location and NLRP3 agonist LPS and MSU were injected at the same time to reduce potential confounders. Sample size was determined based on previous studies and no animals were excluded from the study (Li et al, 1995; Martinon et al, 2006; Man et al, 2017). Mice were maintained at Specific Pathogen Free conditions at University Bern, Switzerland. CD45.1^+^ congenic mice were imported from Charles River (France) and housed for 2 wk before starting the BMT experiments. All animal experiments were approved by the Swiss Federal Veterinary Office under valid authorization (BE39/16). Mice were handled according to Swiss Federal Veterinary Office guidelines under valid authorization. We used the ARRIVE reporting guidelines to report in vivo experiments (du Sert et al, 2020).

### MSU-induced peritonitis model

Mice were injected IP with sterile PBS or MSU (1 mg/mouse) to induce peritonitis. After 12 h mice were euthanized by CO_2_-asphyxiation and the peritoneal cavity was flushed with sterile PBS. The lavage fluid was centrifuged, and supernatants were analysed by ELISA for IL-1β production, and the pelleted cells were analysed for the influx of neutrophils (CD45^+^, Ly-6G^+^, and CD11b^+^) by BD LSRII flow cytometer (BD Biosciences). Data were analysed by using FlowJo (version 9.3.1, TreeStar Inc) software.

### LPS-induced septic shock

Mice were injected IP with LPS (10 mg/kg) and survival rates were monitored.

### Histopathology and BM biopsies

Healthy human controls and inflammatory disease patients BM biopsies were obtained from Swiss MDS Registry. These biopsies were negative for COVID-19 infection. Biopsies were stained with human RNH1 antibody from Prestige Antibodies Sigma-Aldrich (HPA039223). These studies were approved by the competent local human ethics committee (ID:2017-00734).

**Table:** 

Specimen	Clinical information
Healthy control 1	Motorcycle accident and BM without specific changes
Healthy control 2	Bicytopenia and splenomegaly, BM morphologically without specific changes
Patient 1	Metastatic Adenocarcinoma of the lungs with inflammation
Patient 2	Suspicion of mastocytosis, BM without specific changes

### Cells and cell culture media

Mouse BMDMs, iMACs (immortalized macrophages), and human THP-1 cells were used for inflammasome activation experiments.THP-1 cells and iMACs were grown in RPMI 1640 GlutaMAX-I medium (Invitrogen) were supplemented with 10% (vol/vol) FBS (Amimed) and 1% of penicillin and streptomycin (PAA Laboratories) and incubated at 37°C and 5% CO_2_. BMDMs were generated by established protocols. Briefly, total BM cells were isolated from the femur and tibias of mice and differentiated into macrophages for 7 d in complete DMEM medium (500 ml, Thermo Fisher Scientific) supplemented with M-CSF (40 ng/ml) (315-02; Peprotech).

### Reagents and plasmids

We purchased Nigericin (N7143), pdA:dT (poly(dA-dT)•poly(dT-dA)) sodium salt (P0883), Gramicidin A (G5002), PMA (P1585), Actinomycin D and Cycloheximide from Sigma-Aldrich, Ultra-pure flagellin (AG-40B-0025) from Adipogen, ultrapure LPS (LPS-EK), MSU (tlrl-msu), and Pam3Cysk4 (tlrl-pms) from Invivogen.

Cloning of full-length caspase-1 and OMI were amplified by PCR and sub-cloned into the mammalian expression vectors pCR3 in frame with the N-terminal flag tags. Full-length RNH1-Flag construct was described previously (Chennupati et al, 2018). To construct full-length GFP-RNH1 plasmid, a human RNH1-full coding sequence (ORIGEN RC 200082) insert was cloned into the entry vector pENTR4-GFP-C1 (W392-1; Addgene) and then recombined into the destination vector pLenti CMV Blast DEST (706-1; Addgene) plasmid, using the Gateway cloning method.

### Stimulation experiments

All cells were stimulated at a density of 1 × 10^6^ cells per mL as described previously (Allam et al, 2014). For inflammasome studies, THP1 cells were treated with PMA (100 ng/ml) to differentiate into macrophages or BMDMs and iMACs were pre-stimulated for 3 h with 100 ng/ml of ultrapure LPS. Later, cells were stimulated with 1 h with 5 μM of Nigericin or 5 μg/ml of pdA:dT, 30 μg/ml of Gramicidin A, 300 μg/ml MSU and 200 ng/ml of Flagellin. pdA:dT and flagellin were transfected with Lipofectamine 2000 according to the manufacturer’s protocol (Invitrogen). For all conditions, cell-free supernatants were analysed by ELISA for cytokine secretion or cells were lysed for immunoblot analysis.

### CRISPR/CAS9-mediated RNH1-knockout THP1 and iMAC cell line generation

RNH1-KO THP1 cells (human monocytic cell line) were generated as described previously (Chennupati et al, 2018). For RNH1-KO iMAC cells CRISPR sequences targeting exon 2 (RNH1-KO-1) and exon 3 (RNH1-KO-2) of mouse RNH1 were designed and obtained KO cells as described previously (Chennupati et al, 2018). Target exon and the seed sequences preceding the protospacer adjacent motif are the following: RNH1-1 oligo 1- 5′-*CACC*GTCTGA TCCAGCAATACGAAG-3′; RNH1-1 oligo 2—5′-AAACCTTCGTATTGCTGGA TCAGAC-3′; RNH1-2 oligo 1-5′-*CACC*GGATAACCCTATGGGGGACG*C*-3′; RNH1-2 oligo 2-5′-AAACGCGTCCCCCATAGGGTTATCC-3′. All generated THP1 and iMAC clones were tested negative for mycoplasma contamination using MycoAlert Mycoplasma Detection Kit (Cat. no. LT07-318; Lonza). THP1 cells were obtained from ATCC, and iMAC cells were provided by Petr Broz (University of Lausanne) (Broz et al, 2010).

### Generation of stable THP1 cells expressing Flag–RNH1

Flag-RNH1 was further sub-cloned into retroviral vector pMSCVpuro (Clontech). Retroviral vector pMSCVpuro-Flag–RNH1 was co-transfected with the helper plasmids VSV-G and Hit60 into HEK293T cells using PEI transfection reagent. Culture supernatants containing recombinant viral particles were harvested and used to infect THP1 cells. To establish stable cell lines, THP1 cells were selected with puromycin (5 μg ml^−1^) 3 d after infection.

### Generation of transient THP1 cells expressing GFP–RNH1

RNH1-KO THP1 cells were infected with lentiviruses expressing RNH1 (pLenti CMV Blast GFP-RNH1) or the empty vector pLenti CMV-GFP-Blast (659-1; Addgene) as previously described (Papin et al, 2007). After 48 h post-infection, efficiency was monitored by GFP expression, and blasticidin (5 μg/ml) selected THP1 cells were plated for inflammasome assay.

### Generation of stable THP1 cells expressing shRNA-RNH1

Various lentiviral shRNA plasmids against RNH1 were purchased from Sigma-Aldrich and lentivirus was generated as previously described (Chennupati et al, 2018). To establish stable cell lines, THP1 cells were selected with puromycin (5 μg ml^−1^) 3 d after infection.

### Immunoblot analysis

Precipitated media supernatants or cell extracts were analysed by standard immunoblot techniques. The following antibodies were used: anti-human RNH1 (A9), anti-mouse RNH1 (C10), anti-human ASC (Sc-514414), anti-GAPDH (G9, sc-365062) from Santa Cruz Biotechnology; anti-human IL-1β antibody (12242), anti-IκBα, anti-Phospho-IκBα, anti-GFP (2555) from Cell signaling; anti-human caspase-1 (p20, Bally-1), anti-mouse caspase-1 (p20, AG-20B-0042), anti-NLRP3 (Cryo-2), anti-mouse ASC (AG-25B-0006) from Adipogen; anti-mouse IL-1β antibody (AF-401-NA) from R&D; anti-β-Actin from Abcam.

### TLR priming and NF-κB activation

WT and RNH1-KOTHP 1 cells were stimulated with TLR2 agonist Pam3CSK4 (500 ng/ml) at different time points 3, 6, and 14 h or at different doses 100, 500, and 1,000 ng/ml for 3 h. For NF-κ*B* activation, cells were stimulated at 10, 20, 40, 60, and 180 min by Pam3CSK4 (1,000 ng/ml). After stimulation, cell lysates were isolated and analysed via Western blot.

### Caspase-1 expression in HEK293T cells

HEK293T cells were treated with or without MG-132 and transfected with full-length flag-tagged caspase-1 or OMI/HTAR2 plasmids with or without GFP-tagged RNH1 plasmid with the indicated concentration. After 24 h, cells were harvested, and cell lysates were analysed by immunoblot. HEK293T cells routinely tested negative for mycoplasma contamination using MycoAlert Mycoplasma Detection Kit (Cat. no. LT07-318; Lonza). HEK293T cells were obtained from ATCC.

### Cytokine measurement and LDH assay

Cell supernatants were analysed for cytokines and Lactate Dehydrogenase (LDH). Human and mouse IL-1β, TNF, and IL-6 cytokine secretion measured by ELISA according to the manufacturer’s instructions (eBioscience). LDH was measured by colorimetric NAD linked assay by in-house developed kit according OPS Diagnostics instructions.

### Detection of ASC oligomerization by Western blot

We detected ASC oligomerization by Western blot as described previously (Lugrin & Martinon, 2017). Briefly, WT and RNH1-KO THP1 cells were primed with 100 ng of PMA and treated with Nigericin (5 μM) for 1 h and Poly(dA:dT) (5 μg), for 5 h. After stimulation, cells were detached and lysed on ice cold lysis buffer (20 mM Hepes·KOH, pH 7.5, 10 mM KCl, 1.5 mM MgCl_2_, 1 mM EDTA, 1 mM EGTA, and 320 mM sucrose) by syringing 35 times using a 21-G needle. Cell lysates were centrifuged for 8 min at 1,800*g* and supernatants were collected (30 μl of the supernatants were kept aside to check ASC expression in the lysates). Supernatants were further diluted two times with lysis buffer and then centrifuged at 2,000*g* for 5 min. Next, supernatants were diluted with 1 vol of CHAPS buffer (20 mM Hepes·KOH, pH 7.5, 5 mM MgCl_2_, 0.5 mM EGTA, 0.1 mM PMSF, and 0.1% CHAPS) and then centrifuged for 8 min at 5,000*g*. The supernatant was discarded, and the pellet was resuspended in 50 μl of CHAPS buffer supplemented with 4 mM of disuccinimidyl suberate (Thermo Fisher Scientific) and incubated at RT for 30 min. After incubation, samples were centrifuged for 8 min at 5,000*g*, and pellets were resuspended in SDS sample buffer without reducing agents and Western blot was performed.

### Detection of ASC speck formation by confocal microscopy

To further confirm the ASC specks formation, we performed confocal microscopy as described previously (Micco et al, 2016). Briefly, PMA-primed WT and RNH1-KO THP1 cells were seeded on coverslips in 12-well plates before inflammasome activation. Next day, cells were stimulated for 1 h with Nigericin (5 μM) and fixed with 4% (vol/vol) PFA (Applichem) for 20 min at RT. After fixation, cells were washed three times with 1× PBS for 5 min and permeabilized with 0.1% Triton X-100 in 1× PBS for 15 min at RT. Cells were washed with 1× PBS and blocked with 4% (w/vol) BSA for 30 min at RT. After blocking, cells were incubated overnight at 4°C with the primary antibodies against ASC (HASC-71; BioLegend). Cells were washed three times with a washing buffer (1× PBS and 0.025% of Tween20) for 5 min and incubated for 1 h at RT with the Alexa Fluor secondary antibodies (Thermo Fisher Scientific). Cells were washed twice with a washing buffer for 5 min and incubated in with DAPI (1 μg/ml) for 1 min. Again, cells were washed, and coverslips were mounted on glass slides using mounting media (ProLong Gold Antifade). Images were captured with inverted confocal microscope LSM 710 (Zeiss) and analysed with ImageJ across at least 10 fields from three independent experiments.

### Actinomycin D, cycloheximide, and MG-132 chase assays

To study caspase-1 mRNA and protein stability, WT and RNH1-KO THP1 cells were treated with the following inhibitors for 0, 3, and 6 h. Cells were treated with Actinomycin D (10 μg/ml), Cycloheximide (75 μg/ml), proteasomal inhibitor MG-132 (20 μM) to assess transcriptional, translational, and posttranslational changes. After treatment, cells were harvested, and cell lysates were analysed by immunoblot.

### RNA preparation and qRT-PCR

Total RNA was isolated from WT and RNH1-KO THP1 cells using the QIAGEN RNeasy Kit according to the manufacturer’s protocol. Complementary DNA was generated from total RNA as described previously (Allam et al, 2015). The SYBR Green Dye detection system was used for quantitative real-time PCR on Light Cycler 480 (Roche). Controls consisting of ddH_2_O were negative for target and housekeeping genes. The following gene-specific primers (Microsynth) were used. 18S rRNA:5′-GCAATTATTCCCCATGAACG-3′(f), 5′-AGGGCCTCACTAAACCATCC-3′ (r); ASC: 5′-CCT CAG TCG GCA GCC AAG-3′ (f), 5′-CAG CAG CCA CTC AAC GTT TG-3′ (r); CASP1: 5′-TCC TCA GGC TCA GAA GGG AA-3′ (f), 5′-TGC GGC TTG ACT TGT CCA T-3′ (r); RNH1: 5′-GATCTGGGAGTGTGGCATCA-3′ (f), 5′-CTGCAGGACTTCACCCACAG-3′ (r).

### COVID-19 pseudovirus infection

The SARS-CoV-2 pseudoviral particles expressing COVID-19 spike protein pGBW m4137384: S protein was purchased from Addgene (149543) and the virus particles were produced as described previously (Hoffmann et al, 2020; Nie et al, 2020). WT and RNH1-KO THP1 cells were treated with PMA (100 ng/ml) to differentiate into macrophages and infected with SARS-CoV-2 pseudovirus at MOI of 0.25 and 1 for 24 h. Infection efficiency was monitored by measuring GFP signal using fluorescent microscope (Leica DMI 400 B).

### COVID-19 patient samples

We analysed a total of 28 COVID-19 patients samples from a previously published study (n = 13) (Spinetti et al, 2020), and with addition of new patients to the cohort (n = 15). We isolated protein from buffy coats of COVID-19 patients and analysed RNH1 expression by Western blot. The present study is secondary analysis from the main COVID-project (NCT04510012) (Spinetti et al, 2020), and these results were not part of the pre-specified analyses for the main project. The study was performed in accordance with the “Declaration of Helsinki” and approved by the Kantonale Ethikkommission KEK, Bern, Switzerland, Nr. 2020-00877.

### COVID-19 tissue microarray samples

Post-mortem (PM) tissues were collected as part of the ethically approved LoST-SoCC study in accordance with the “Declaration of Helsinki” and Human Tissue Authority legislation. The study was approved by the Newcastle North Tyneside 1 research ethics committee 19/NE/0336, IRAS 193937. Non–COVID-19 causes of death were all non-respiratory, non-infectious, obtained from PMs that were done before the pandemic, and lungs were morphologically within normal limits. The original FFPE tissue blocks were taken from representative samples at post-mortem, formalin fixed and processed as per standard diagnostic blocks. For tissue microarray (TMA) construction, representative 3 mm tissue cores were cored out of FFPE tissue blocks and re-blocked within a 4 × 4 format TMA, sectioned and mounted onto glass slides. Slides were dehydrated and endogenous peroxides quenched. Antigen retrieval was done using TRIS buffer pH9 boiled for 15 min. Slides were incubated with a permeabilization buffer (0.3% Triton-X100 in PBS) then blocking buffer (10% FBS in PBS). Anti-RNH1 (HPA039223; Prestige Antibodies Sigma-Aldrich) was added overnight at 1:500, followed by anti-rabbit secondary, ABC amplification and DAB detection (both Thermo Fisher Scientific). Slides were then counterstained with Meyer’s haematoxylin and coverslips applied with aqueous mounting agent and slides imaged using Zeiss axioscan Z1. Images were analysed using Zen Lite software (Zeiss).

### Sequence conservation analysis

Human RNH1 protein sequence was used as a query to perform Blastp with human refseq proteins. From Blast output, selected protein sequences (excluding the isoforms and model refseq proteins) were aligned using MAFFT multiple sequence aligner (Katoh & Standley, 2013). Sequence conservation analysis was performed on IQ-Tree webserver (http://iqtree.cibiv.univie.ac.at/) using ultrafast bootstrap analysis with 1,000 number of bootstrap alignment (Katoh & Standley, 2013). Circular tree was visualized using Interactive Tree of Life (iTOL) (Letunic & Bork, 2019). For structural alignment of protein domains, the domain information of selected proteins was taken from Uniprot and represented using Illustrator for Biological Sequences (IBS) tool (Liu et al, 2015). Sequences used for multiple sequence alignment: NP_002930.2_RNH1; NP_653288.1_NLRP12; NP_789792.1_NLRP14; NP_703148.4_NLRP5; NP_001120933.2_NLRP3; NP_659444.2_NLRP11; NP_789790.2_NLRP9; NP_001167553.1_NLRP2; NP_604393.2_NLRP4; NP_789780.2_NLRP13; NP_001120727.1_NLRP7; NP_789781.2_NLRP8; NP_127497.1_NLRP1; NP_001264057.1_LRRC31; NP_001263629.1_NLRP6; NP_006083.1_NOD1; NP_079101.4_PODNL1; NP_060110.4_CARMIL1.

### Statistical analysis

All the data were represented as mean ± SEM. Comparison between two groups was performed by a two-tailed *t* test. A value of *P* < 0.05 was considered statistically significant. To compare the survival in LPS model, log-rank (Mantel–Cox) test was performed. For correlation studies, Spearman’s rank correlation statistical test was performed. Statistical analyses for COVID-19 patients’ data were performed with R. All other statistical analyses were calculated using GraphPad Prism version 9.

## Data Availability

Plasmids and transgenic mice are available upon request. Please write to the lead contact: allam.ramanjaneyulu@dbmr.unibe.ch.

## Supplementary Material

Reviewer comments
