# Supplementary material for: LRR-protein RNH1 dampens the inflammasome activation and is associated with COVID-19 severity
Source: Life Sci Alliance. 2022 Mar 7;5(6):e202101226. doi: 10.26508/lsa.202101226 (PMC8922048; doi:10.26508/lsa.202101226)
Supplement: Supplementary file 1 [file LSA-2021-01226_SdataF1_F2_F3_F4_F5_F6_FS1_FS2_FS3_FS4_FS5.pdf]

Figure 1.E

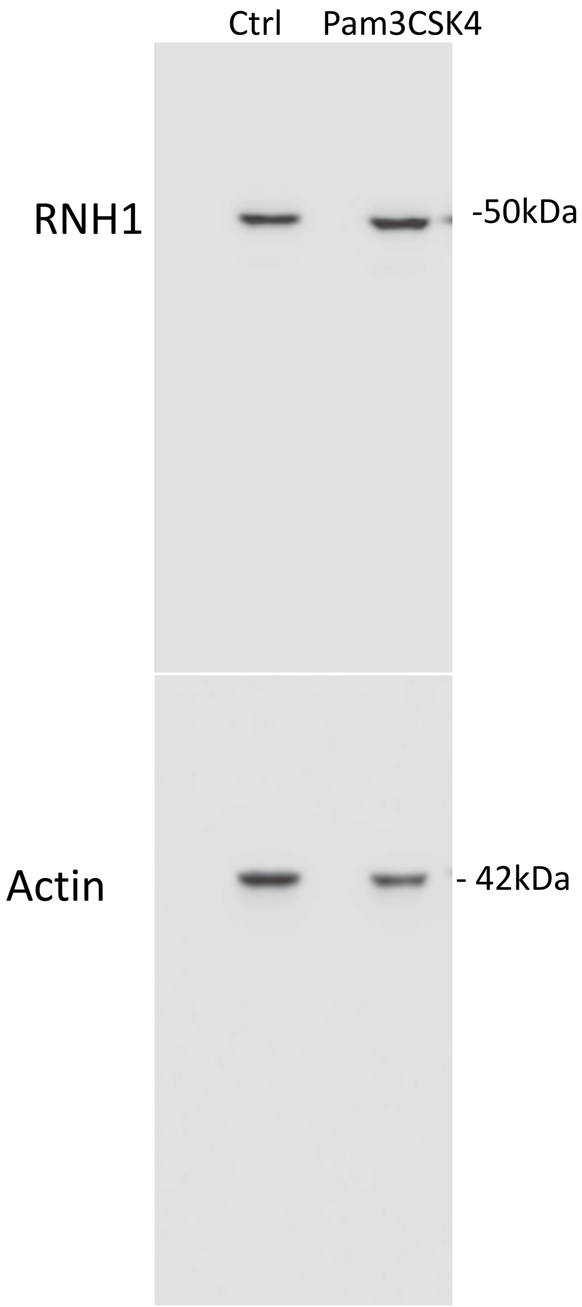

Figure 1.F

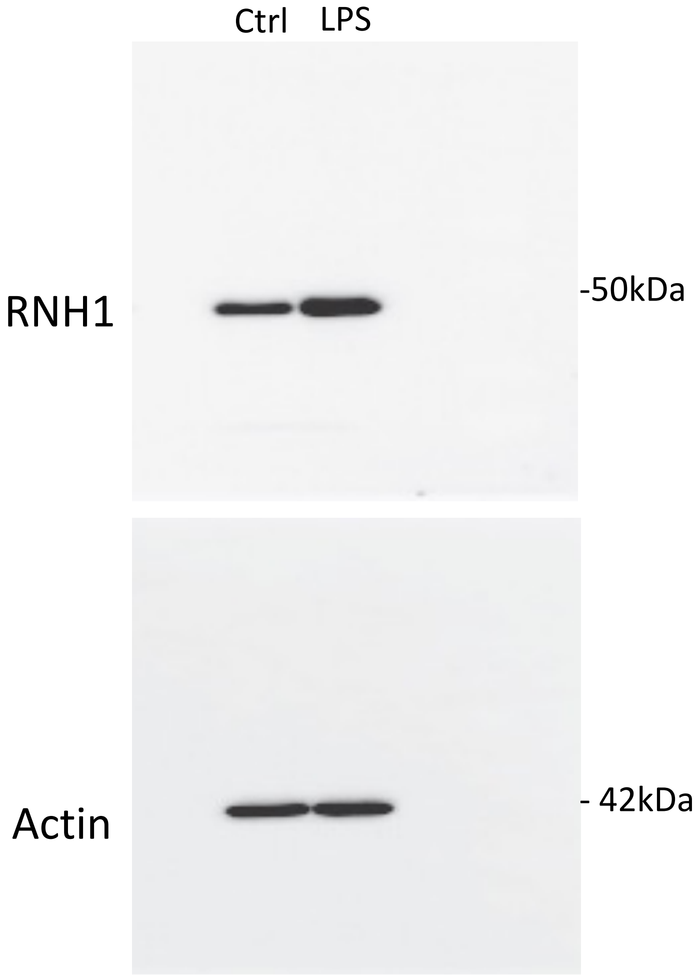

Figure.2B

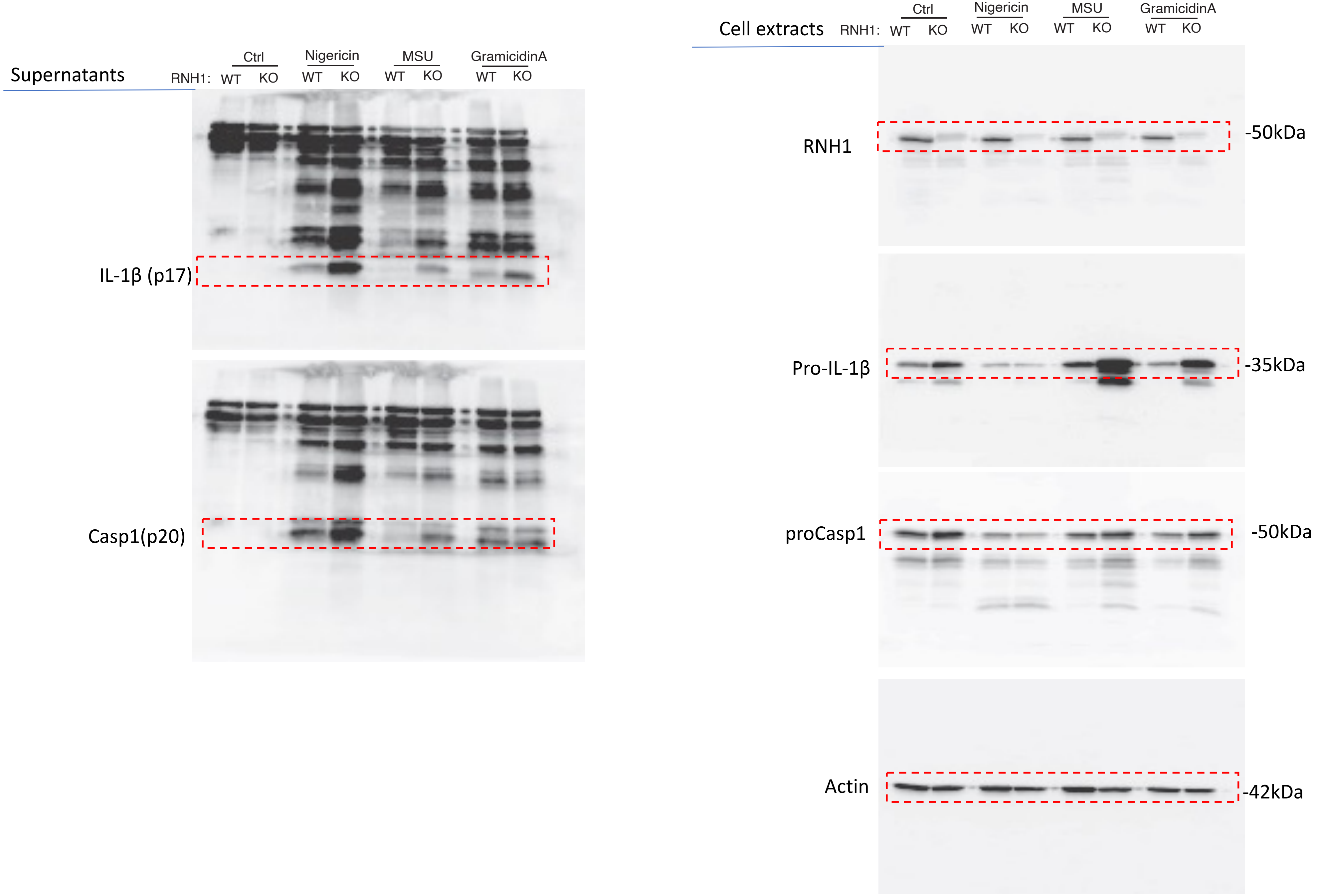

Supernatants
RNH1: WT KO WT KO WT KO WT KO
Ctrl
Nigericin
MSU
GramicidinA
IL-1β (p17)
Casp1(p20)
Cell extracts
RNH1: WT KO WT KO WT KO WT KO
RNH1
-50kDa
Pro-IL-1β
-35kDa
proCasp1
-50kDa
Actin
-42kDa

Figure.2D

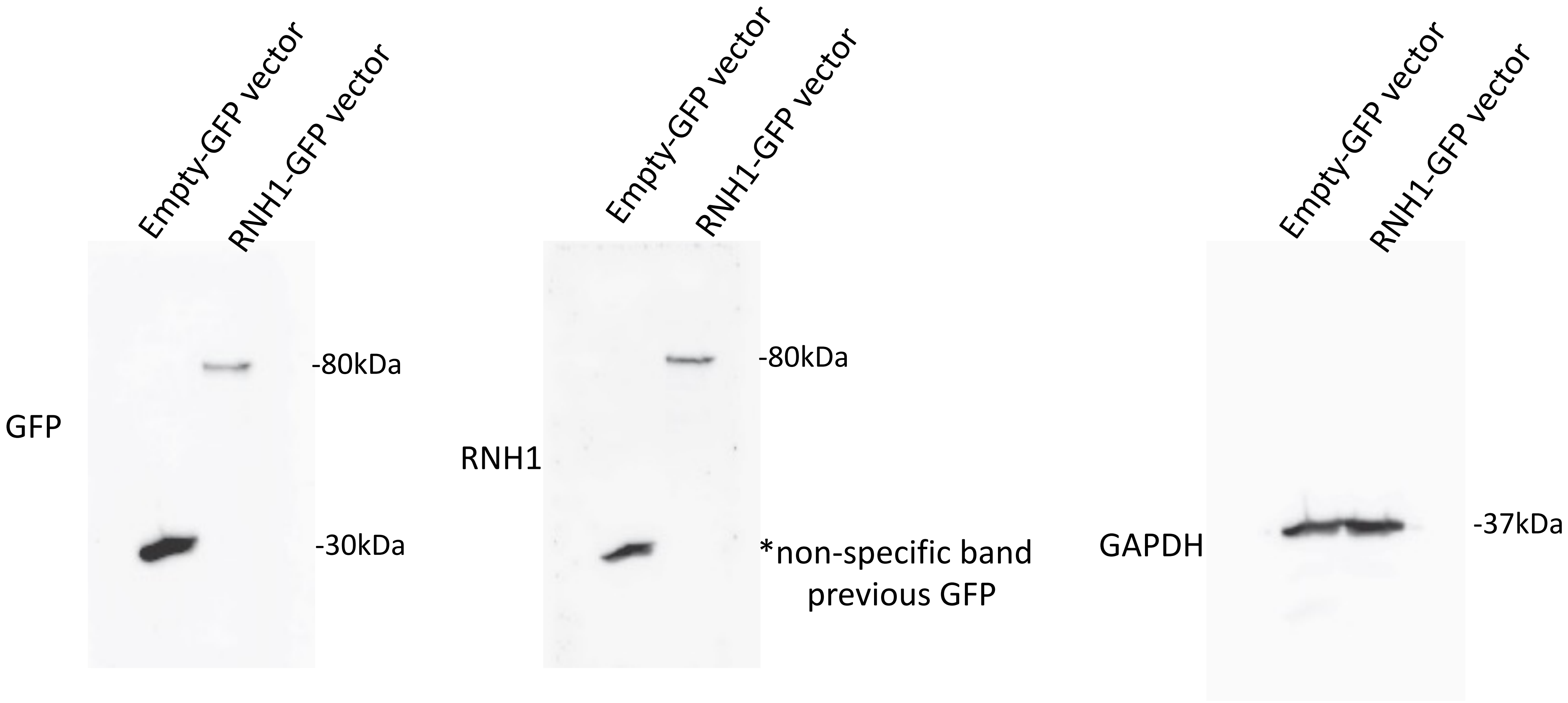
Empty-GFP vector
RNH1-GFP vector
GFP
-80kDa
-30kDa
Empty-GFP vector
RNH1-GFP vector
RNH1
-80kDa
*non-specific band
previous GFP
Empty-GFP vector
RNH1-GFP vector
GAPDH
-37kDa

Figure.2F

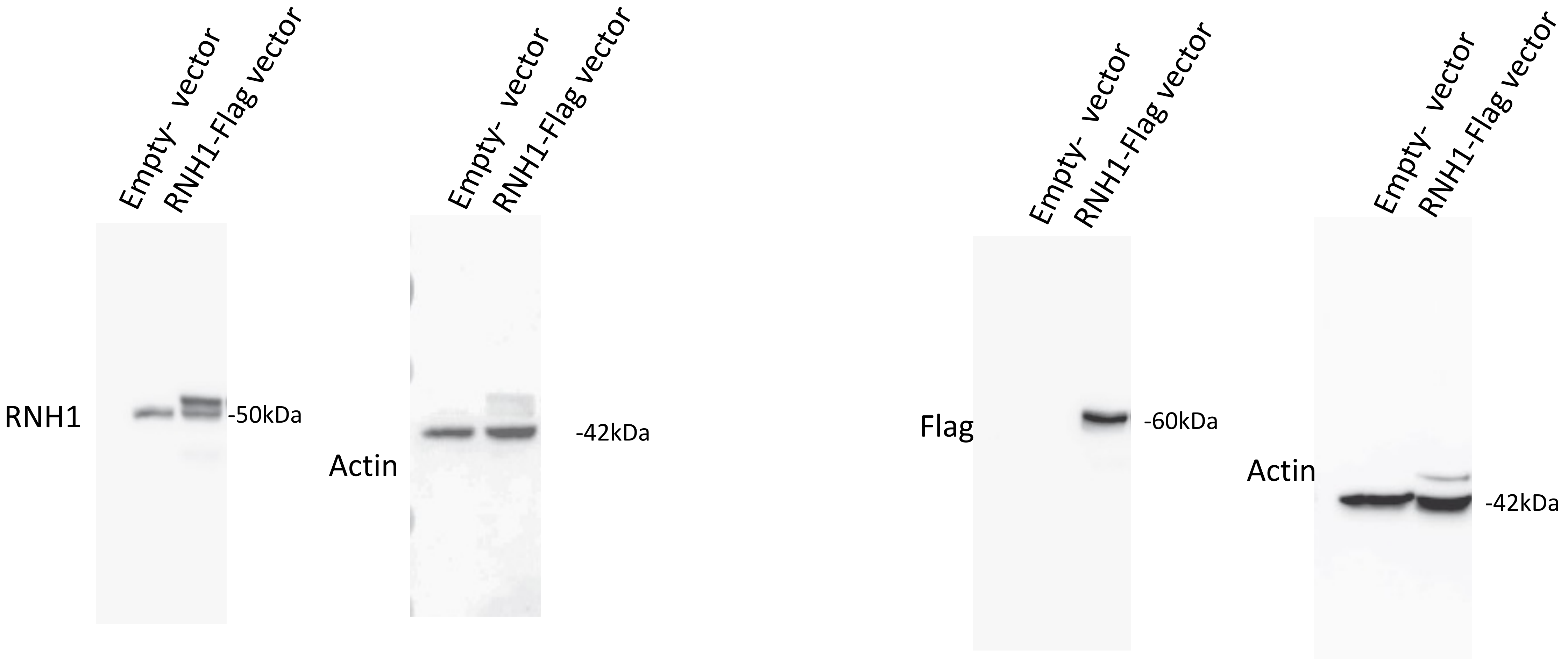

Empty- vector
RNH1-Flag vector
RNH1
-50kDa
Empty- vector
RNH1-Flag vector
Actin
-42kDa
Empty- vector
RNH1-Flag vector
Flag
-60kDa
Empty- vector
RNH1-Flag vector
Actin
-42kDa

Figure.3A

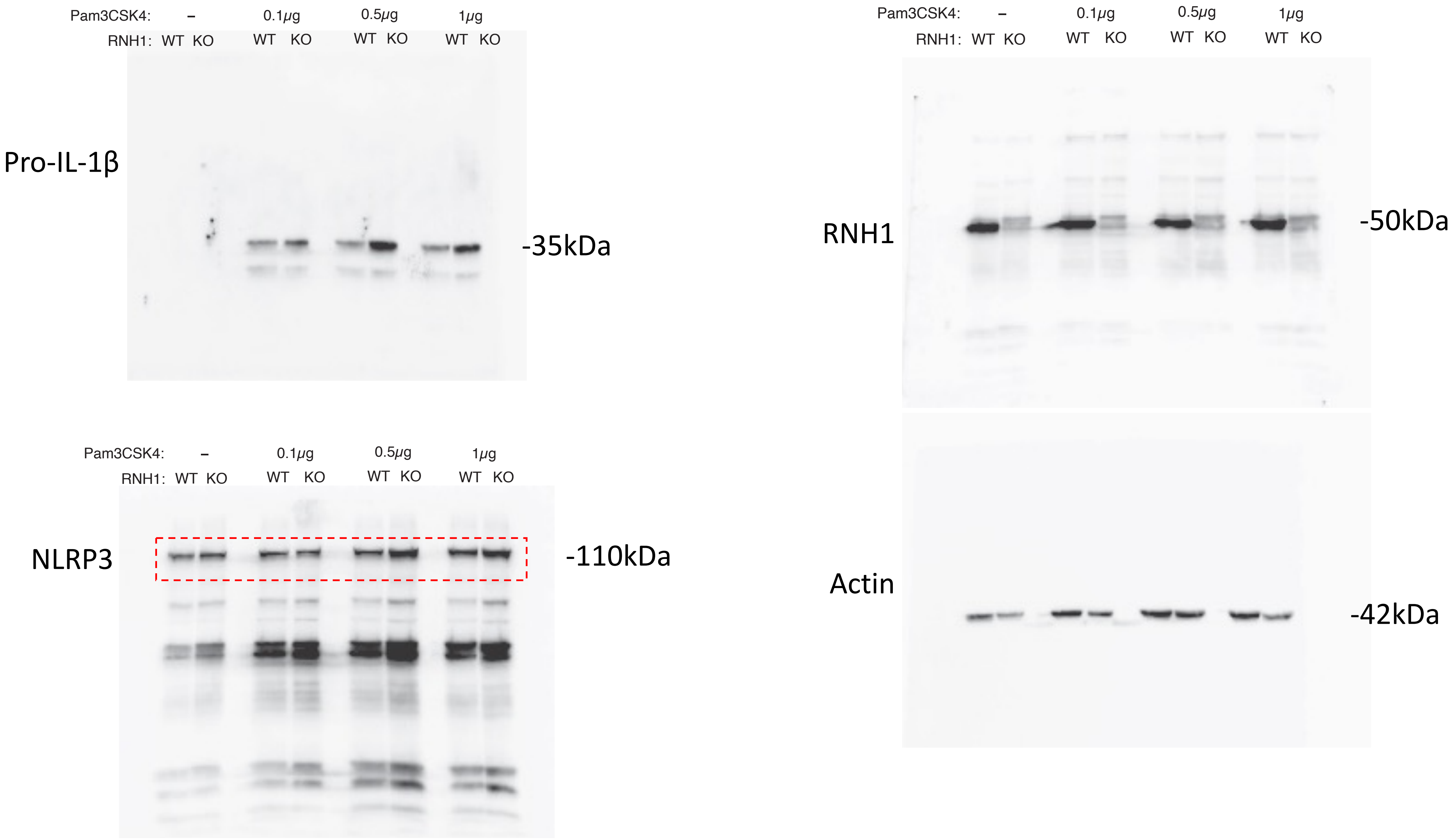
Pam3CSK4: – 0.1μg 0.5μg 1μg
RNH1: WT KO WT KO WT KO WT KO
Pro-IL-1β
-35kDa
NLRP3
-110kDa
RNH1
-50kDa
Actin
-42kDa

Figure.3B

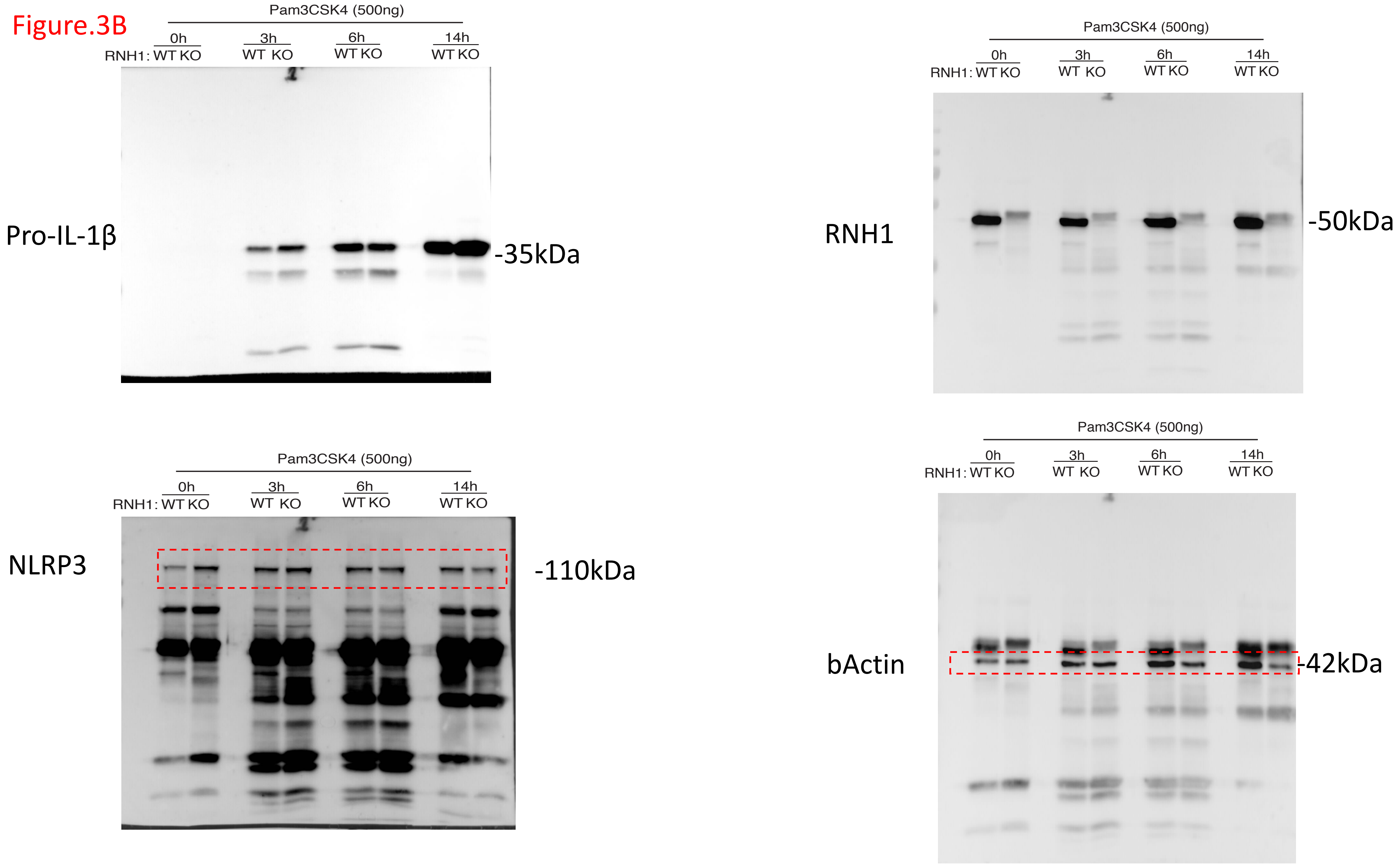

Pam3CSK4 (500ng)
0h
3h
6h
14h
RNH1: WT KO
WT KO
WT KO
WT KO
Pro-IL-1β
-35kDa
RNH1
-50kDa
NLRP3
-110kDa
bActin
-42kDa

Figure.3C

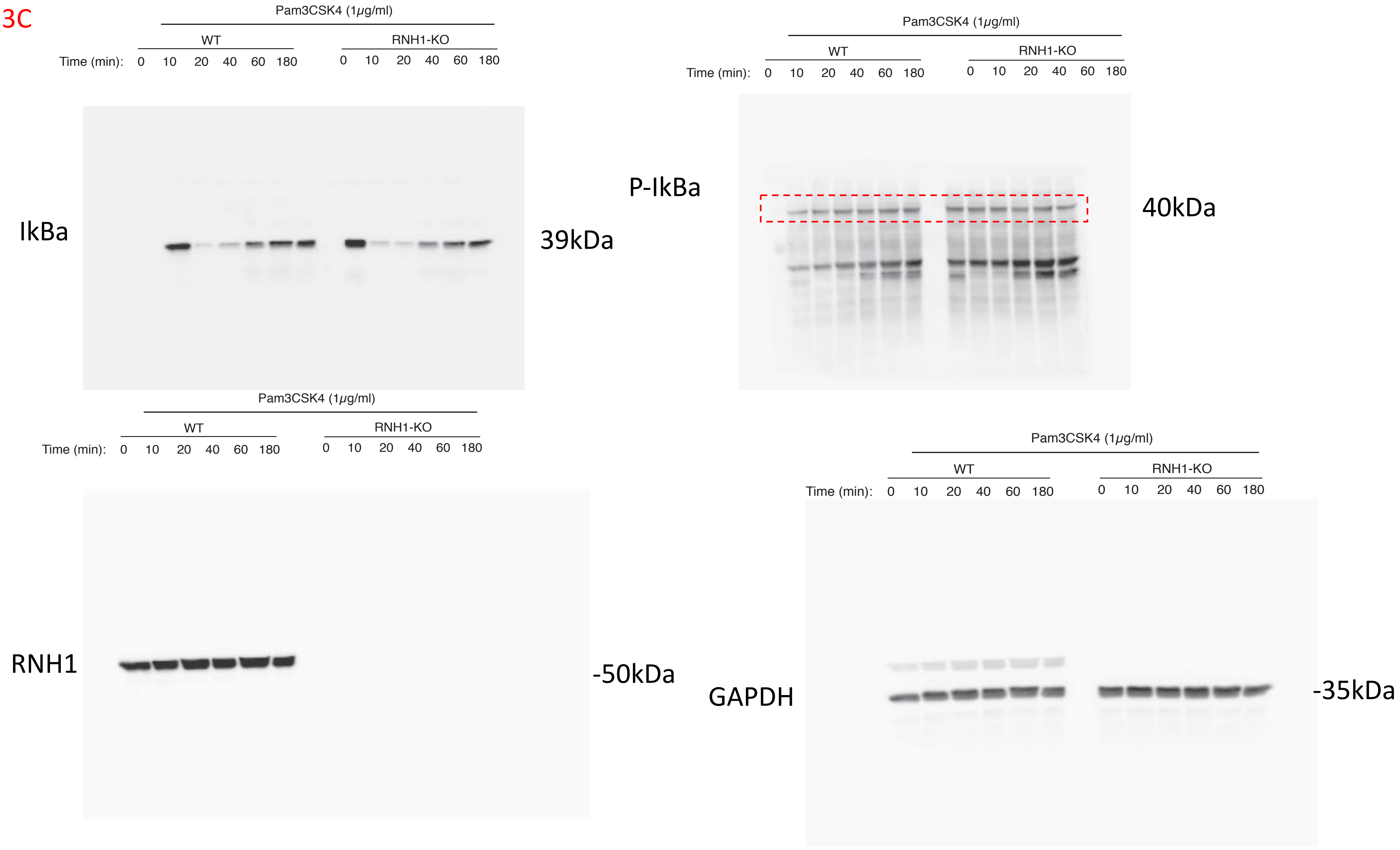

Pam3CSK4 (1μg/ml)
WT
RNH1-KO
Time (min): 0 10 20 40 60 180 0 10 20 40 60 180
IkBa
39kDa
Pam3CSK4 (1μg/ml)
WT
RNH1-KO
Time (min): 0 10 20 40 60 180 0 10 20 40 60 180
P-IkBa
40kDa
Pam3CSK4 (1μg/ml)
WT
RNH1-KO
Time (min): 0 10 20 40 60 180 0 10 20 40 60 180
RNH1
-50kDa
Pam3CSK4 (1μg/ml)
WT
RNH1-KO
Time (min): 0 10 20 40 60 180 0 10 20 40 60 180
GAPDH
-35kDa

Figure.3G

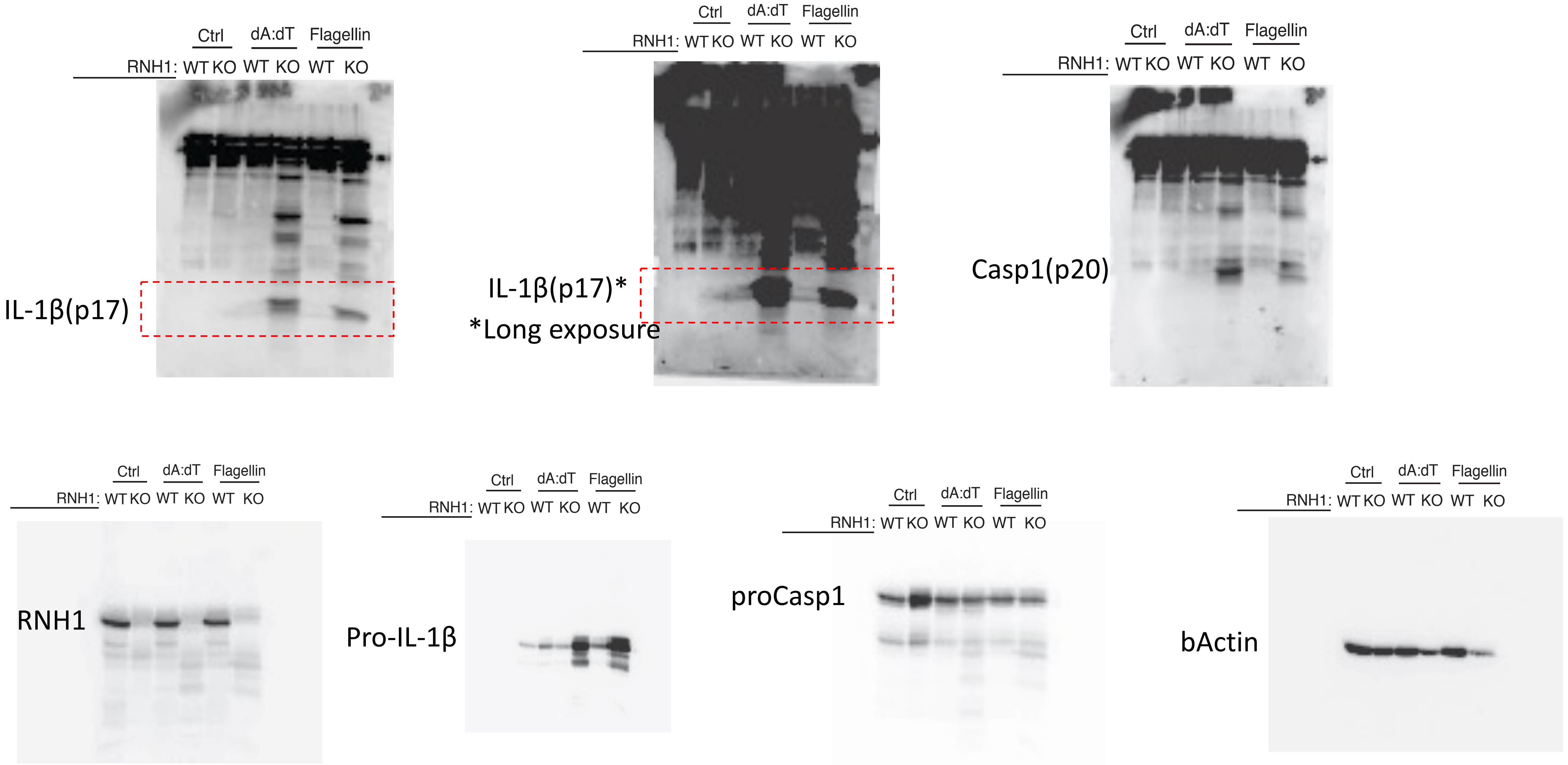
Ctrl
dA:dT
Flagellin
RNH1: WT KO WT KO WT KO
IL-1β(p17)
Ctrl
dA:dT
Flagellin
RNH1: WT KO WT KO WT KO
IL-1β(p17)*
*Long exposure
Ctrl
dA:dT
Flagellin
RNH1: WT KO WT KO WT KO
Casp1(p20)
Ctrl
dA:dT
Flagellin
RNH1: WT KO WT KO WT KO
RNH1
Ctrl
dA:dT
Flagellin
RNH1: WT KO WT KO WT KO
Pro-IL-1β
Ctrl
dA:dT
Flagellin
RNH1: WT KO WT KO WT KO
proCasp1
Ctrl
dA:dT
Flagellin
RNH1: WT KO WT KO WT KO
bActin

Figure.4A

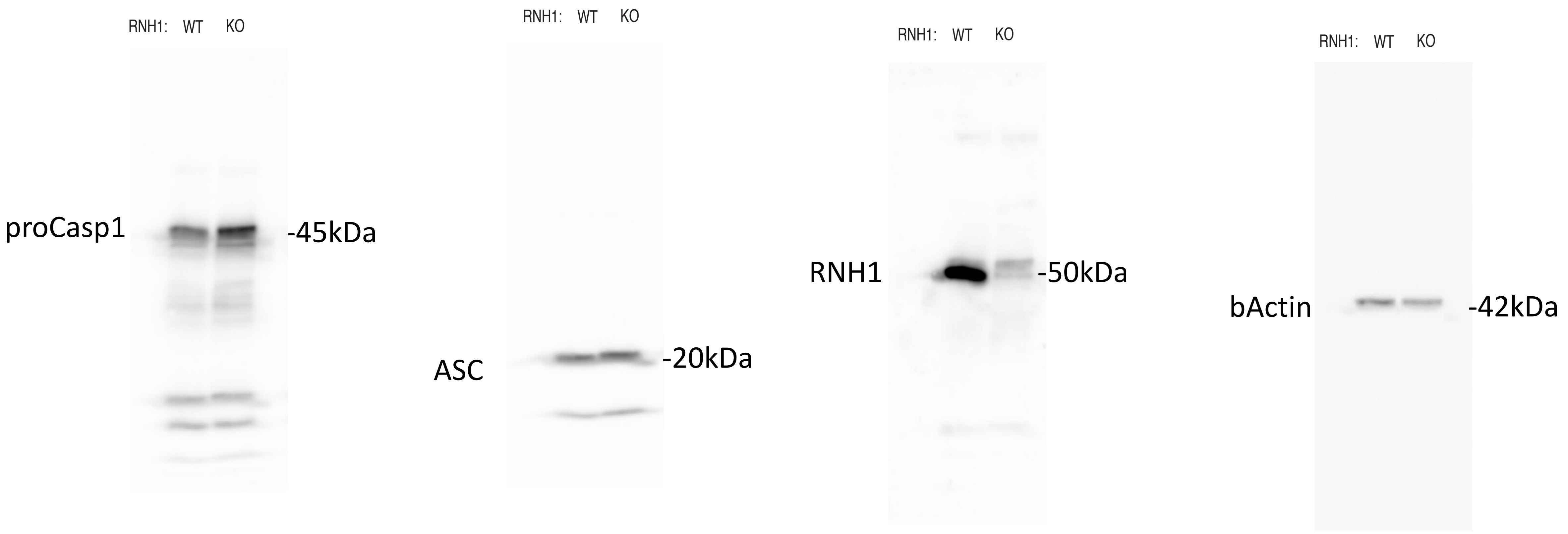
RNH1: WT KO
proCasp1
-45kDa
RNH1: WT KO
ASC
-20kDa
RNH1: WT KO
RNH1
-50kDa
RNH1: WT KO
bActin
-42kDa

Figure.4C

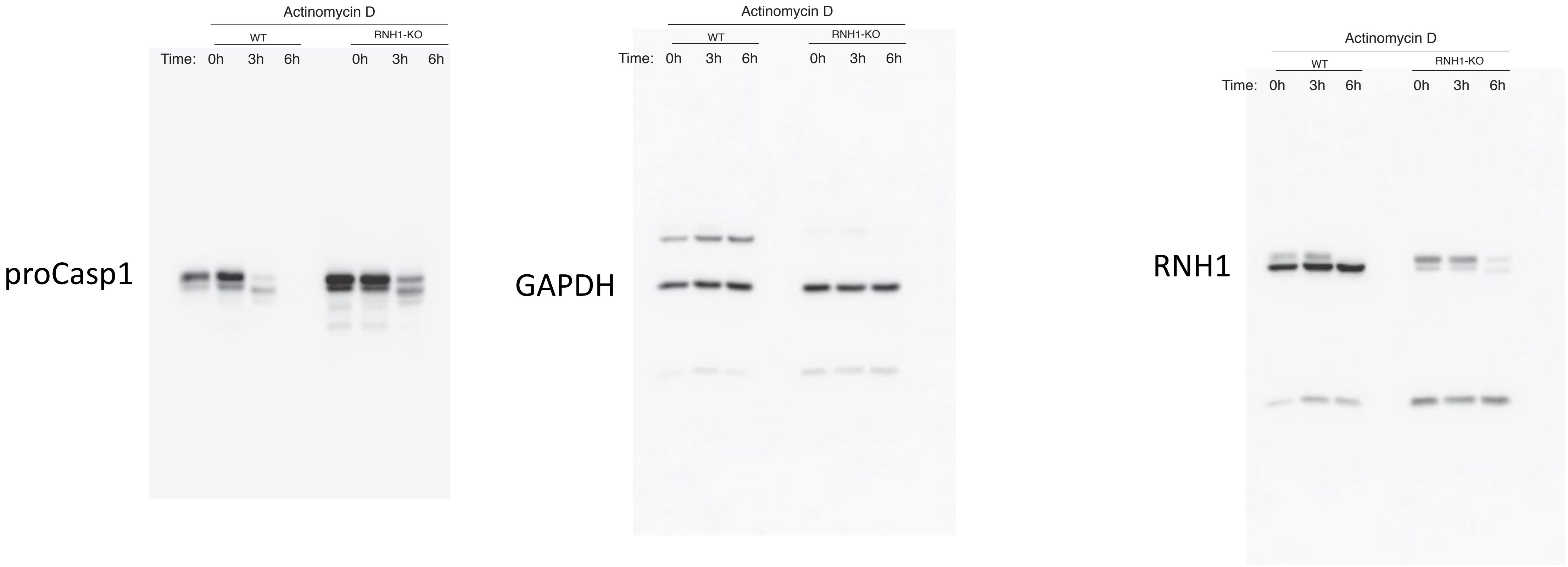

Actinomycin D
WT
RNH1-KO
Time: 0h 3h 6h 0h 3h 6h
proCasp1
Actinomycin D
WT
RNH1-KO
Time: 0h 3h 6h 0h 3h 6h
GAPDH
Actinomycin D
WT
RNH1-KO
Time: 0h 3h 6h 0h 3h 6h
RNH1

Figure.4D

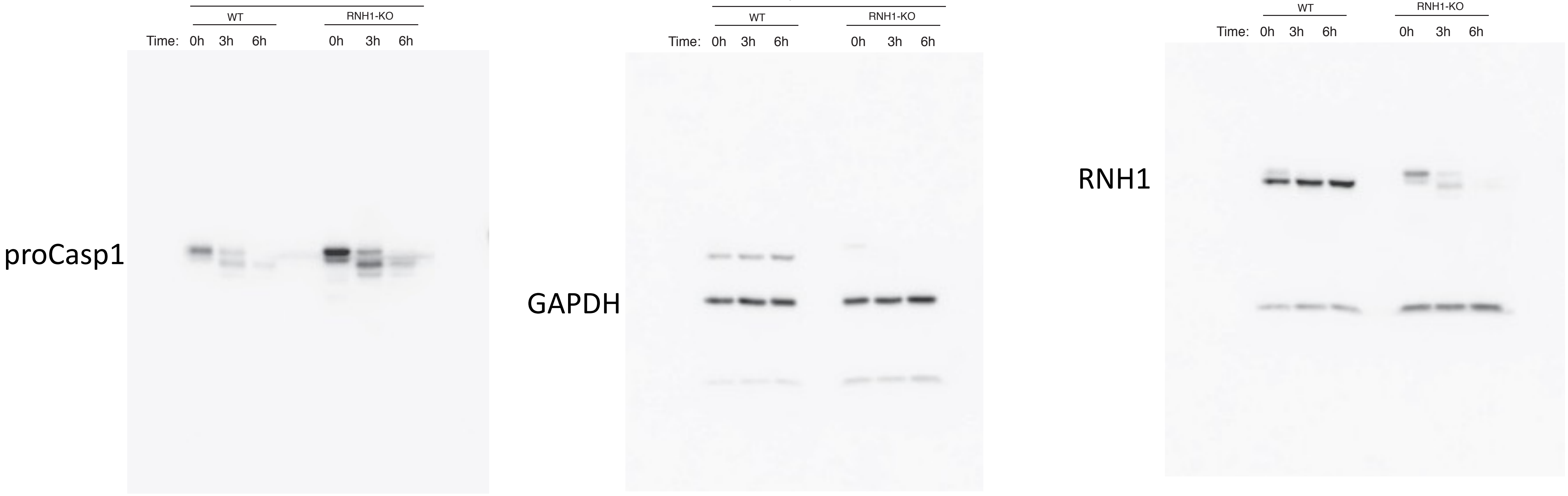

Cycloheximide
WT
RNH1-KO
Time: 0h 3h 6h 0h 3h 6h
proCasp1
Cycloheximide
WT
RNH1-KO
Time: 0h 3h 6h 0h 3h 6h
GAPDH
Cycloheximide
WT
RNH1-KO
Time: 0h 3h 6h 0h 3h 6h
RNH1

Figure.4E

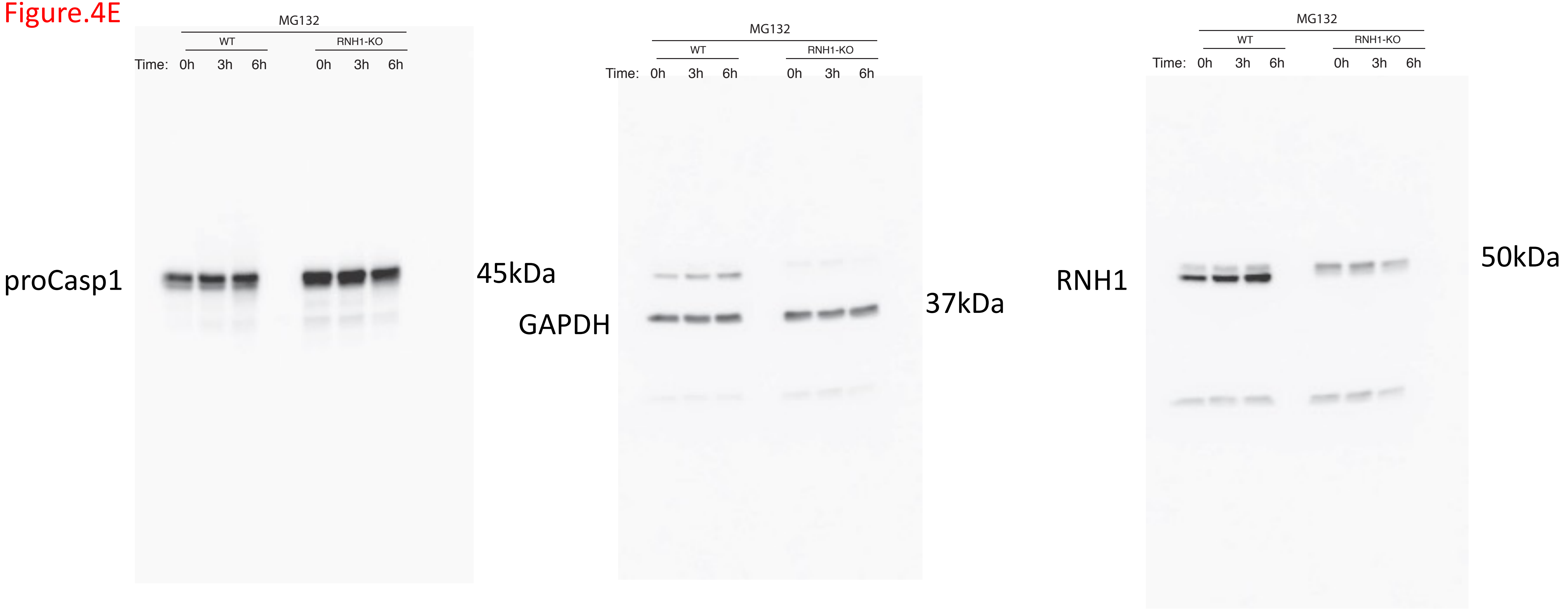
MG132
WT
RNH1-KO
Time: 0h 3h 6h 0h 3h 6h
proCasp1
45kDa
GAPDH
37kDa
RNH1
50kDa

Figure.4F

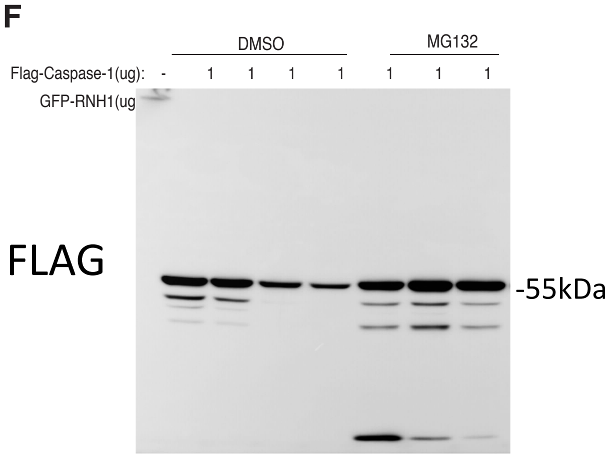
F
DMSO
MG132
Flag-Caspase-1(ug): - 1 1 1 1 1 1 1
GFP-RNH1(ug
FLAG
-55kDa

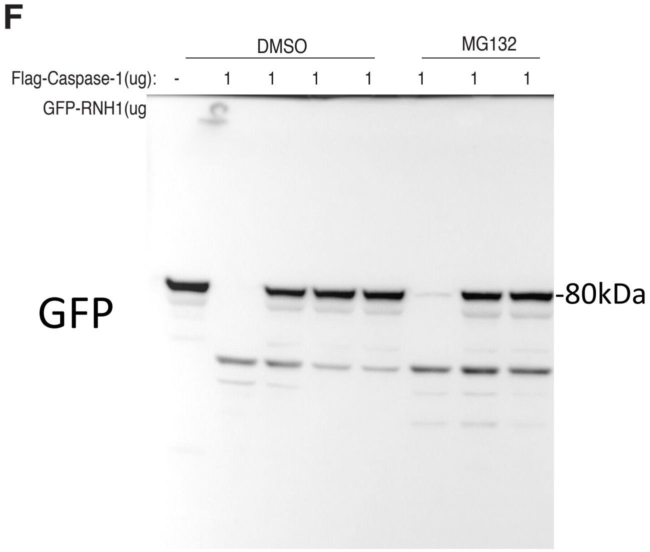
F
DMSO
MG132
Flag-Caspase-1(ug): - 1 1 1 1 1 1 1
GFP-RNH1(ug
GFP
-80kDa

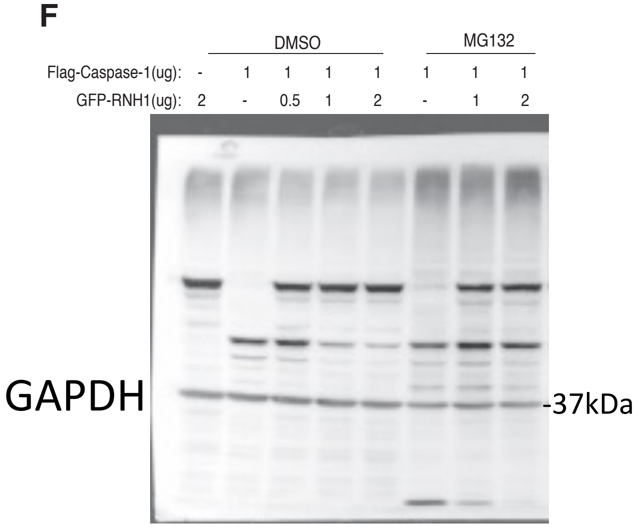
F
DMSO
MG132
Flag-Caspase-1(ug): - 1 1 1 1 1 1 1
GFP-RNH1(ug): 2 - 0.5 1 2 - 1 2
GAPDH
-37kDa

Figure.4G

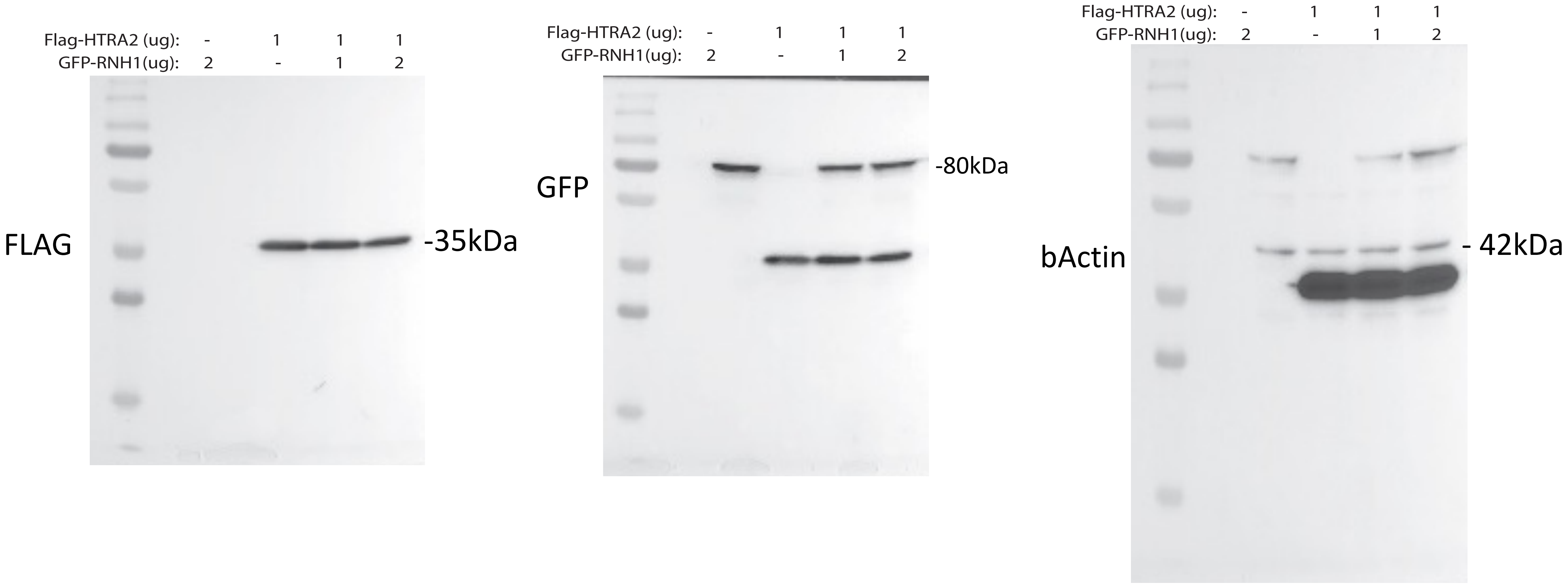
Flag-HTRA2 (ug): - 1 1 1
GFP-RNH1(ug): 2 - 1 2
FLAG
-35kDa
Flag-HTRA2 (ug): - 1 1 1
GFP-RNH1(ug): 2 - 1 2
GFP
-80kDa
Flag-HTRA2 (ug): - 1 1 1
GFP-RNH1(ug): 2 - 1 2
bActin
- 42kDa

Fig 5C

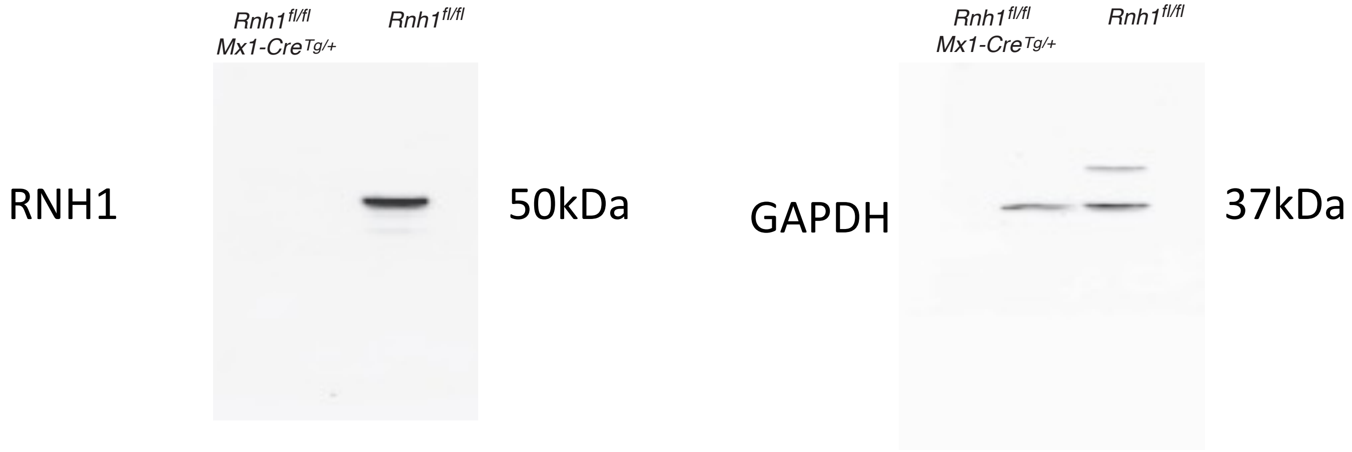
Rnh1 fl/fl
Mx1-Cre Tg/+
Rnh1 fl/fl
RNH1
50kDa
Rnh1 fl/fl
Mx1-Cre Tg/+
Rnh1 fl/fl
GAPDH
37kDa

Fig 5E

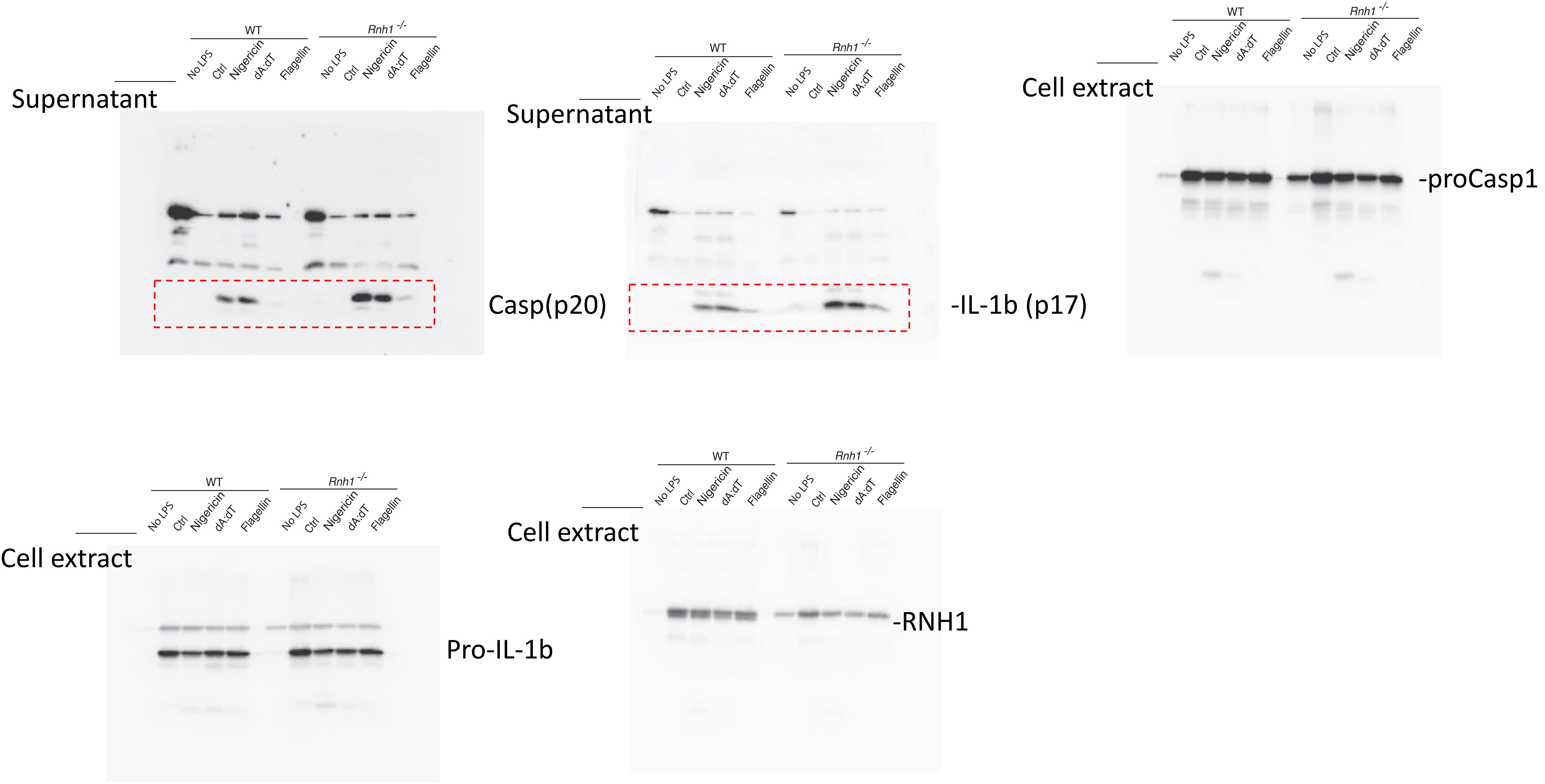

WT
Rnh1 -/-
No LPS
Ctrl
Nigericin
dA:dT
Flagellin
Supernatant
Casp(p20)
-IL-1b (p17)
Cell extract
-proCasp1
Pro-IL-1b
-RNH1

Fig 6A

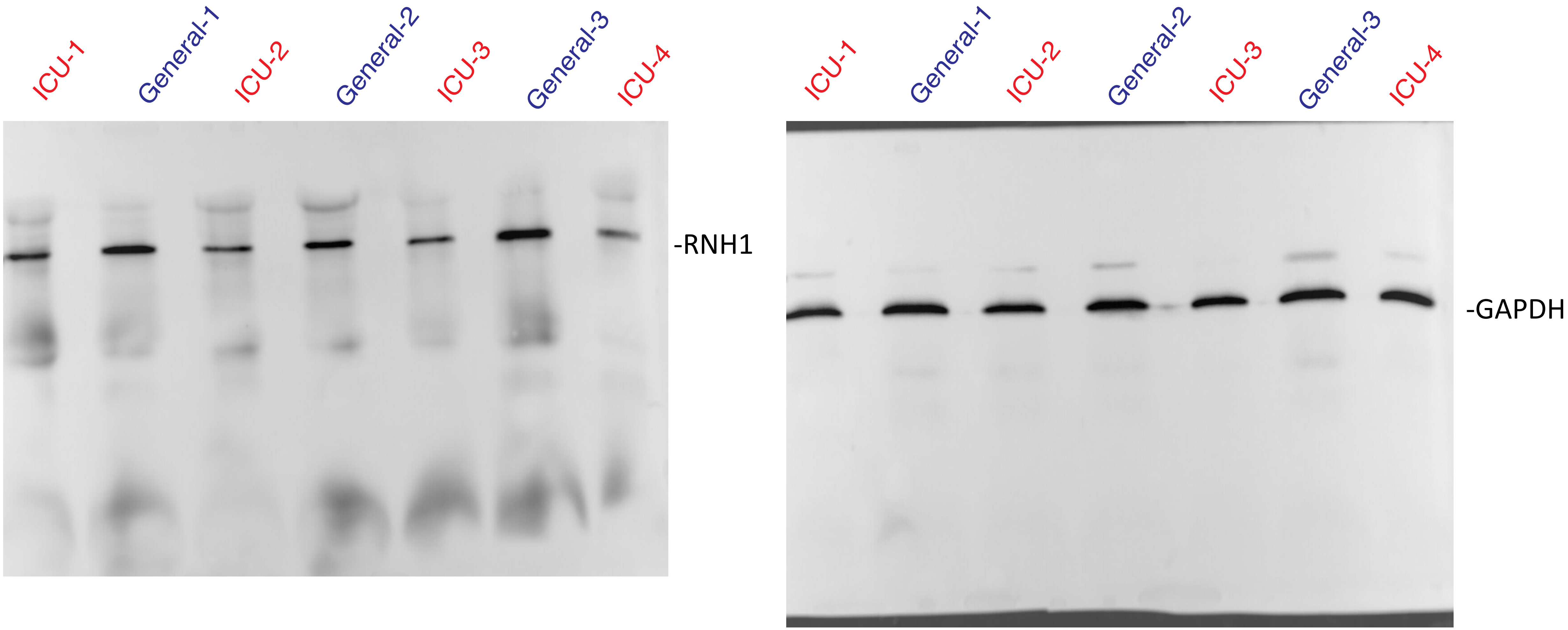
ICU-1
General-1
ICU-2
General-2
ICU-3
General-3
ICU-4
-RNH1
ICU-1
General-1
ICU-2
General-2
ICU-3
General-3
ICU-4
-GAPDH

Sup Fig 1B

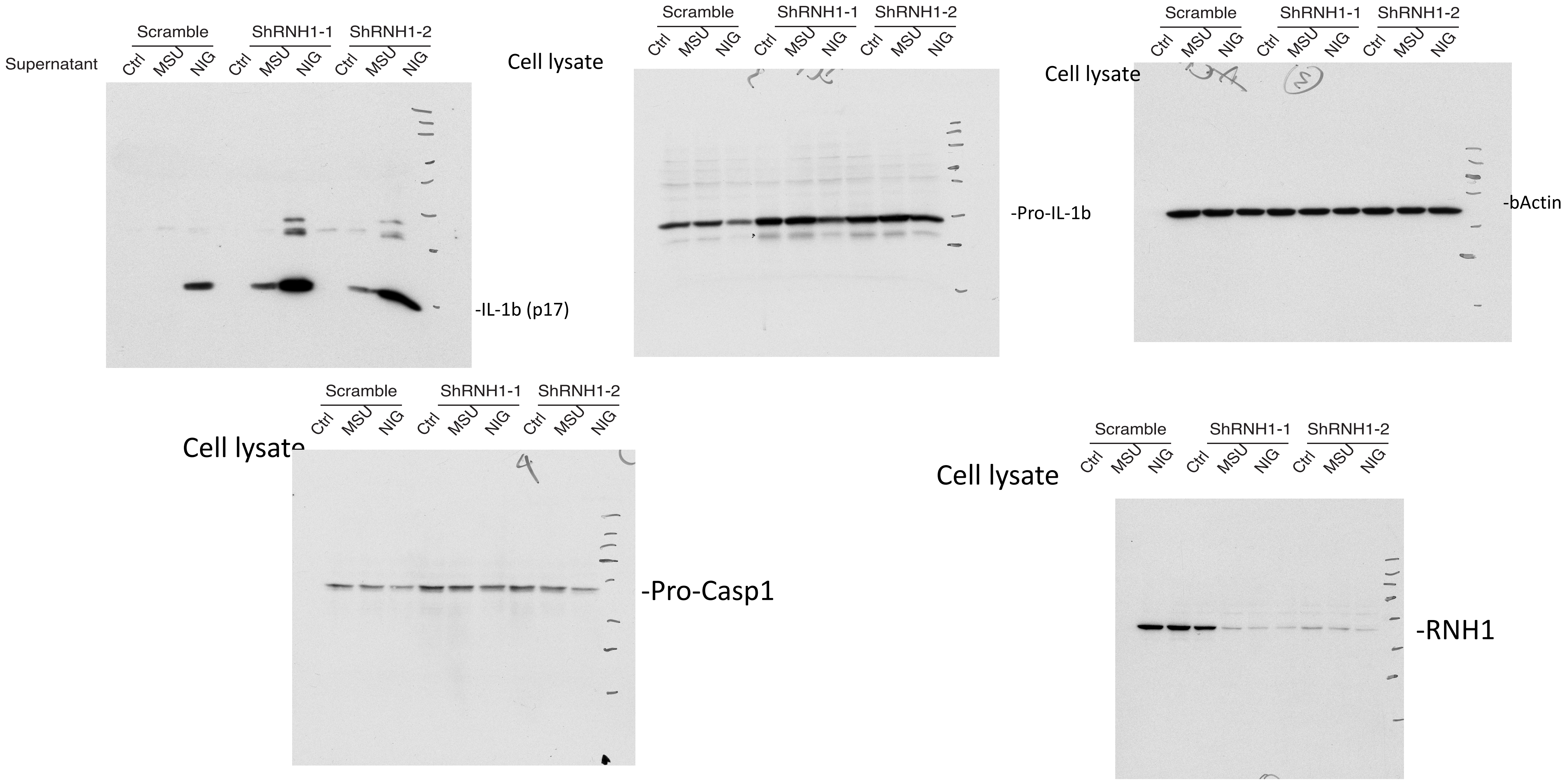
Supernatant
Scramble
ShRNH1-1
ShRNH1-2
Ctrl
MSU
NIG
-IL-1b (p17)
Cell lysate
-Pro-IL-1b
Cell lysate
-bActin
Cell lysate
-Pro-Casp1
Cell lysate
-RNH1

Sup Fig 3A

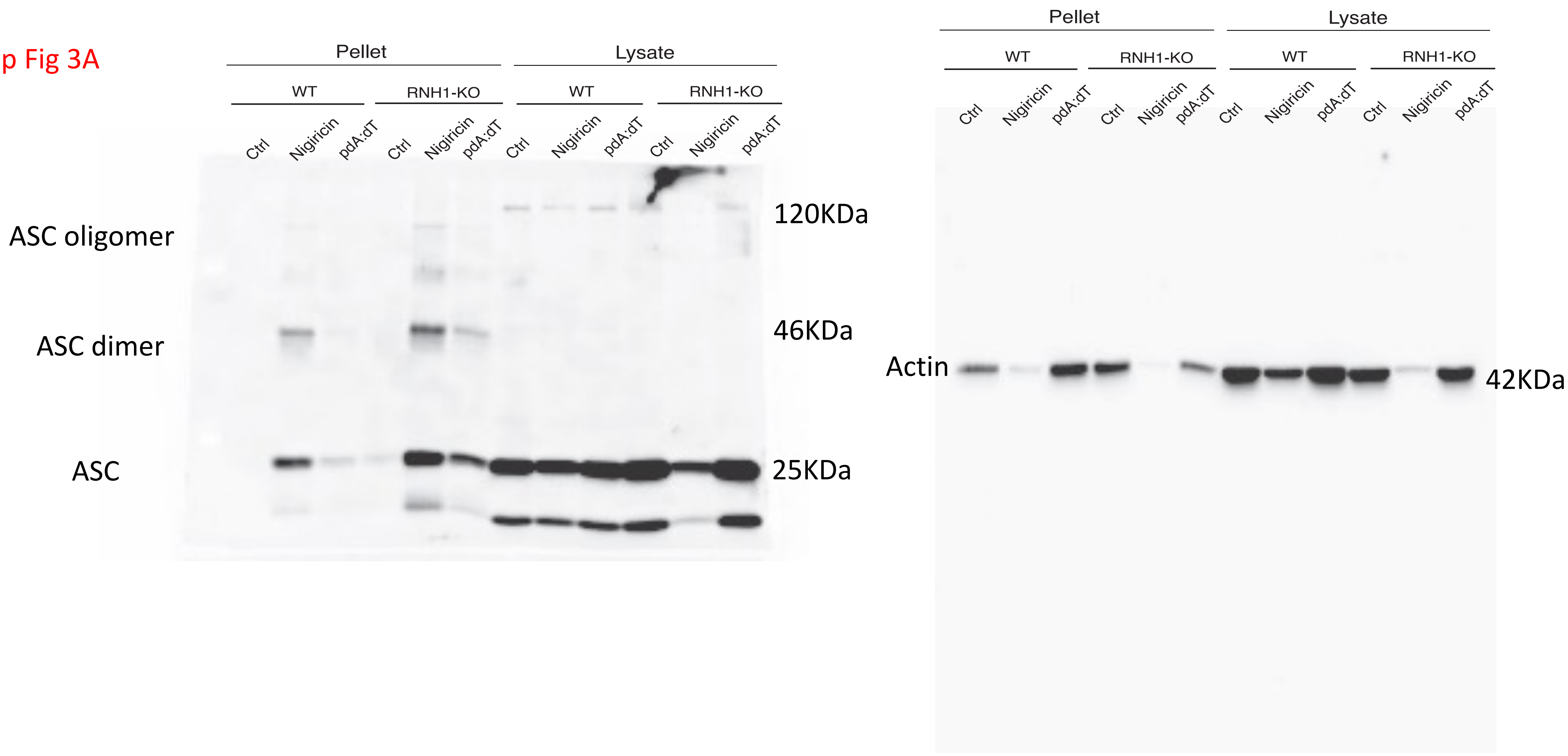

Pellet
Lysate
WT
RNH1-KO
WT
RNH1-KO
Ctrl
Nigiricin
pdA:dT
ASC oligomer
ASC dimer
ASC
120KDa
46KDa
25KDa
Pellet
Lysate
WT
RNH1-KO
WT
RNH1-KO
Ctrl
Nigiricin
pdA:dT
Actin
42KDa

Sup Fig 4

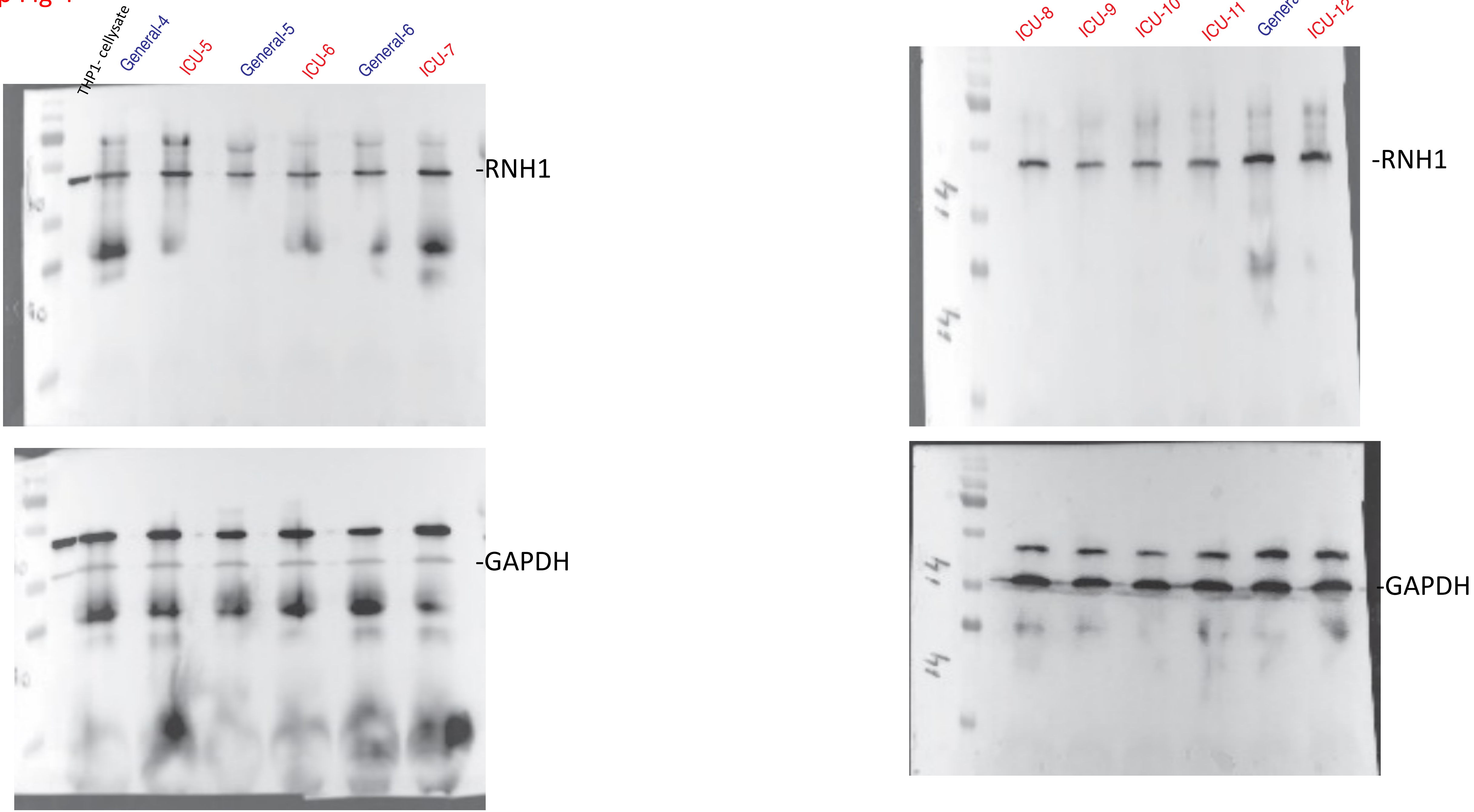
THP1- cellysate
General-4
ICU-5
General-5
ICU-6
General-6
ICU-7
-RNH1
-GAPDH
ICU-8
ICU-9
ICU-10
ICU-11
General-7
ICU-12
-RNH1
-GAPDH

Sup Fig 4

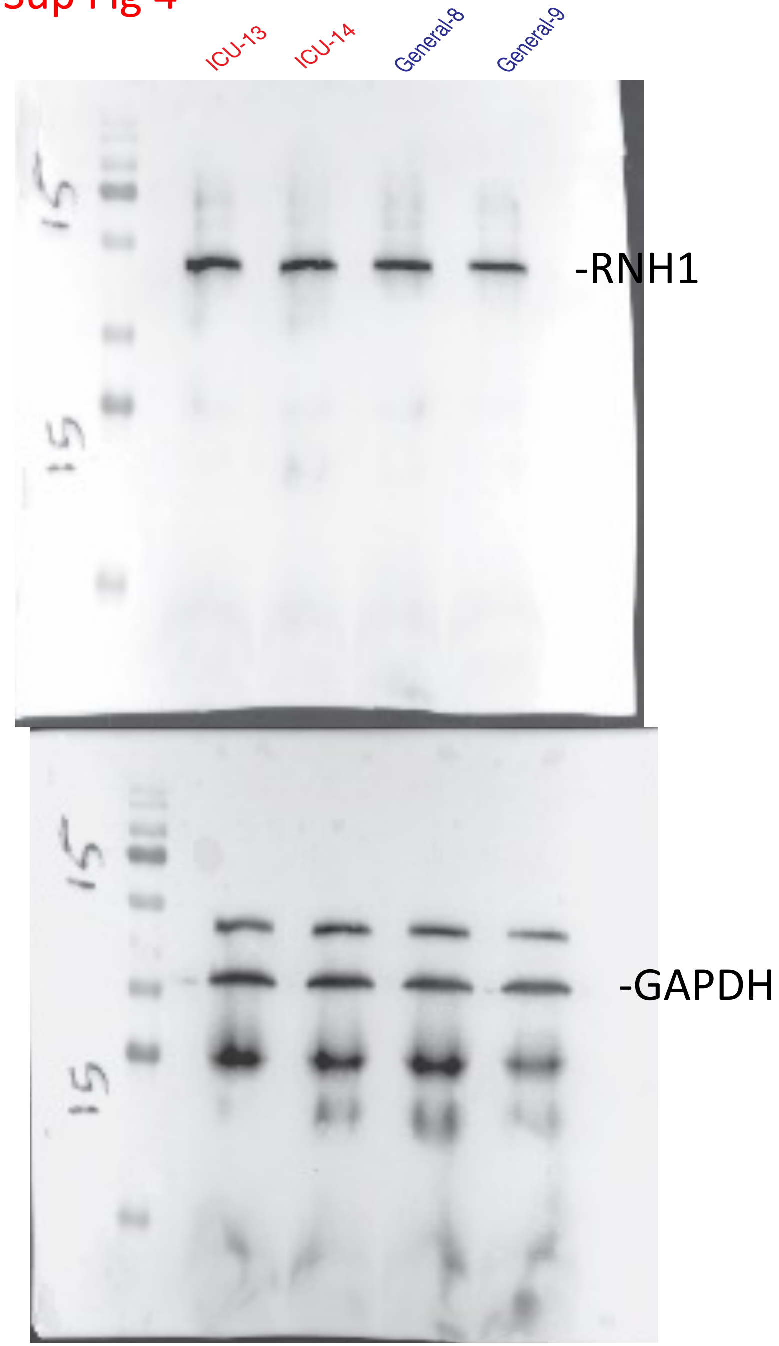

ICU-13
ICU-14
General-8
General-9
15
15
-RNH1
15
15
-GAPDH

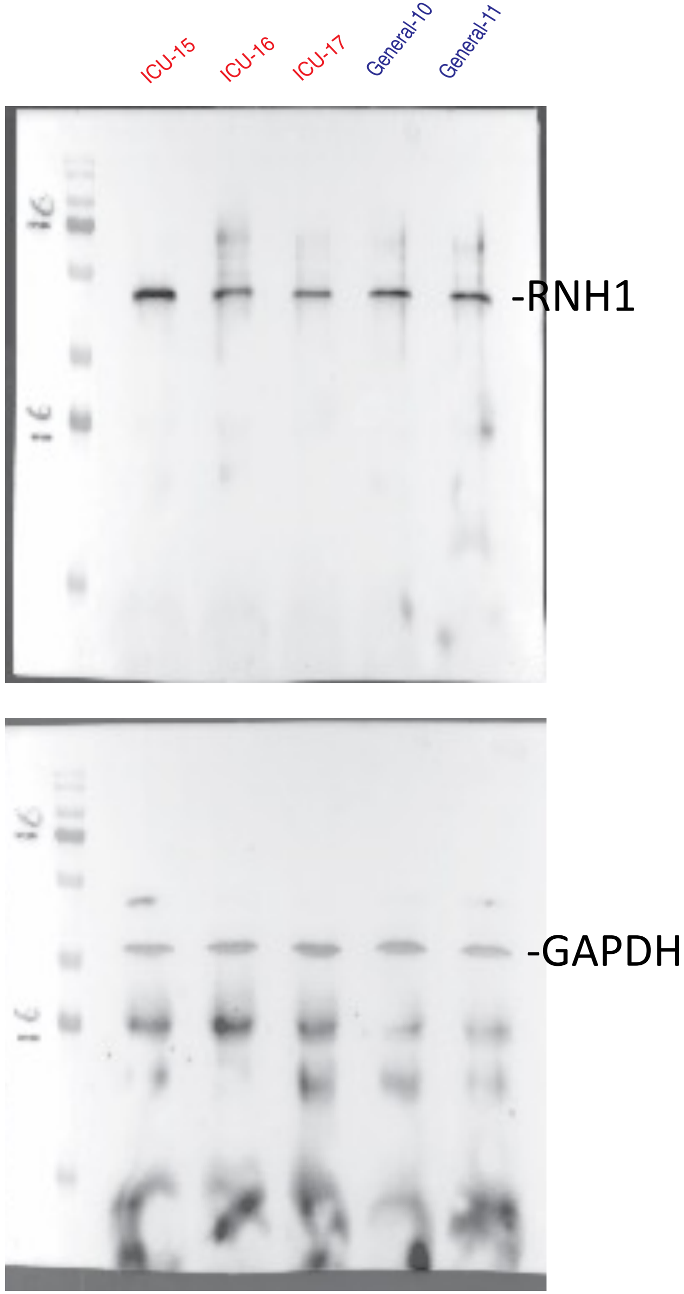

ICU-15
ICU-16
ICU-17
General-10
General-11
-RNH1
-GAPDH
